# A Critical Review and Meta-Analysis of Impacts of Per- and Polyfluorinated Substances on the Brain and Behavior

**DOI:** 10.3389/ftox.2022.881584

**Published:** 2022-04-11

**Authors:** Hannah M. Starnes, Kylie D. Rock, Thomas W. Jackson, Scott M. Belcher

**Affiliations:** Center for Environmental and Health Effects of PFAS, Department of Biological Sciences, North Carolina State University, Raleigh, NC, United States

**Keywords:** behavior, blood-brain barrier, brain, development, fluorochemical, neurotoxicity, persistent, PFAS

## Abstract

Per- and polyfluoroalkyl substances (PFAS) are a class of structurally diverse synthetic organic chemicals that are chemically stable, resistant to degradation, and persistent in terrestrial and aquatic environments. Widespread use of PFAS in industrial processing and manufacturing over the last 70 years has led to global contamination of built and natural environments. The brain is a lipid rich and highly vascularized organ composed of long-lived neurons and glial cells that are especially vulnerable to the impacts of persistent and lipophilic toxicants. Generally, PFAS partition to protein-rich tissues of the body, primarily the liver and blood, but are also detected in the brains of humans, wildlife, and laboratory animals. Here we review factors impacting the absorption, distribution, and accumulation of PFAS in the brain, and currently available evidence for neurotoxic impacts defined by disruption of neurochemical, neurophysiological, and behavioral endpoints. Emphasis is placed on the neurotoxic potential of exposures during critical periods of development and in sensitive populations, and factors that may exacerbate neurotoxicity of PFAS. While limitations and inconsistencies across studies exist, the available body of evidence suggests that the neurobehavioral impacts of long-chain PFAS exposures during development are more pronounced than impacts resulting from exposure during adulthood. There is a paucity of experimental studies evaluating neurobehavioral and molecular mechanisms of short-chain PFAS, and even greater data gaps in the analysis of neurotoxicity for PFAS outside of the perfluoroalkyl acids. Whereas most experimental studies were focused on acute and subchronic impacts resulting from high dose exposures to a single PFAS congener, more realistic exposures for humans and wildlife are mixtures exposures that are relatively chronic and low dose in nature. Our evaluation of the available human epidemiological, experimental, and wildlife data also indicates heightened accumulation of perfluoroalkyl acids in the brain after environmental exposure, in comparison to the experimental studies. These findings highlight the need for additional experimental analysis of neurodevelopmental impacts of environmentally relevant concentrations and complex mixtures of PFAS.

## Introduction

### What are PFAS?

**FIGURE 1:**
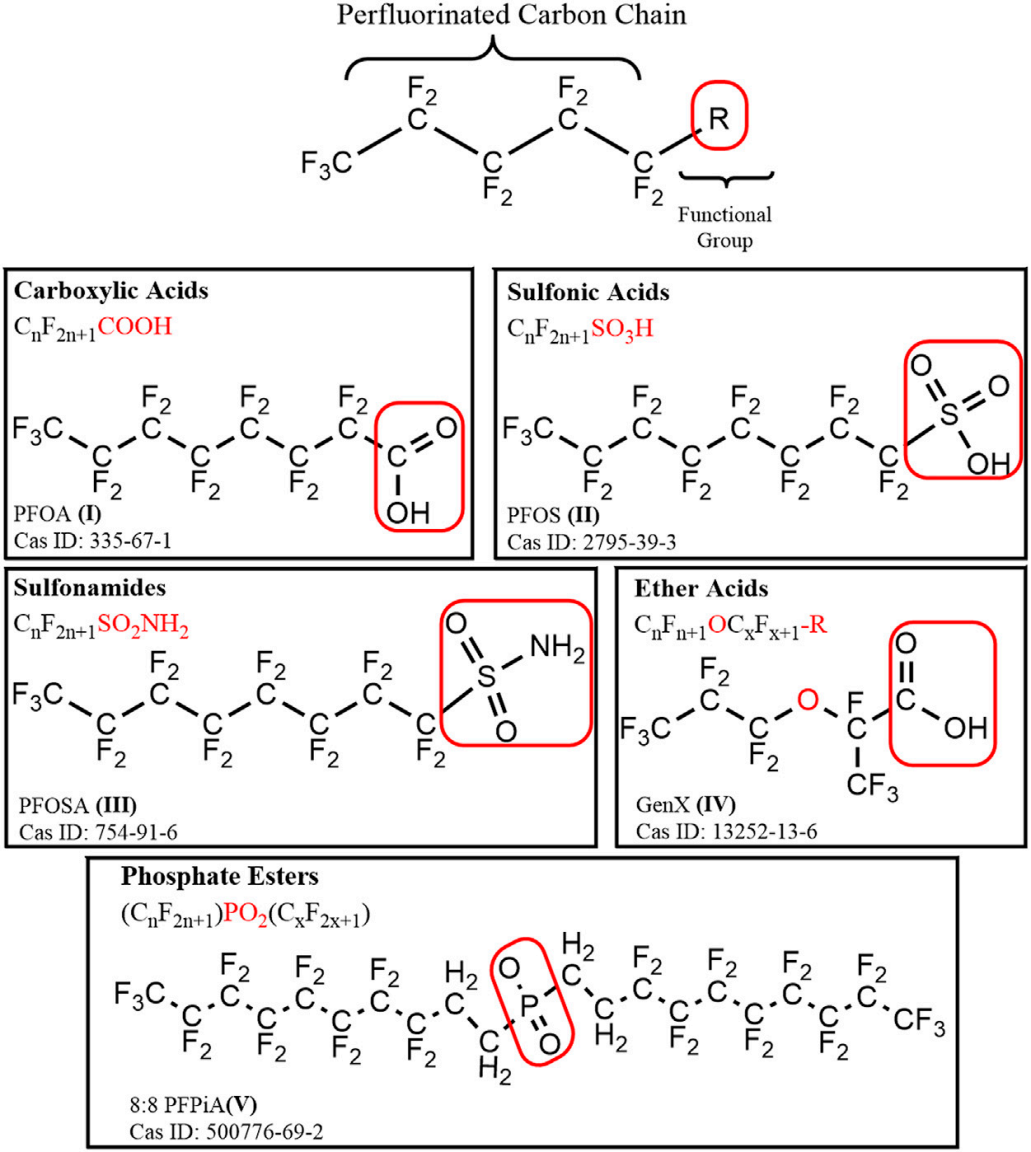
A general structural formula for perfluoroalkyl substance (PFAS), containing a hydrophobic perfluorinated alkyl tail, and a hydrophilic functional (R) group outlined in a red box. Example compounds are depicted for each of the major chemical classes of PFAS discussed: carboxylic acids, sulfonic acids, sulfonamides, ether acids, and phosphate esters.

Per- and polyfluorinated alkyl substances (PFAS) are a large group of structurally diverse aliphatic compounds, distinguished by a fully (per-) or partially (poly-) fluorinated chain of carbon atoms, often connected to at least one functional group (Figure 1; Supplementary Table S1; OECD, 2021). Most PFAS are amphiphilic compounds, due to the hydrophobic properties of their fluorinated alkyl “tail” and hydrophilic properties of their functional groups. Following the synthesis of the first known PFAS compound, polytetrafluoroethylene (PFTE) in 1938, production of PFAS quickly ramped up after their discovery, as the surfactant, oil-repellent, and water-repellent qualities they possess made them valuable for a variety of applications and commercial products (Plunkett, 1986; Lehmler, 2005; Renner, 2006; Lindstrom et al., 2011). Those products include, but are not limited to, non-stick coatings, waterproof apparel, fire-fighting foams, paints, textiles, carpets, cleaning products, and lubricants. While PFAS have proven to be useful for a wide variety of applications, they have also received attention for a multitude of undesirable qualities related to their chemical stability, toxicity, persistence, high mobility, and wide-spread use.

Contamination of air, surface, and ground water results from PFAS release by manufacturing processes, use at industrial sites, wastewater processing, fire-fighting training, and at various life cycle stages of PFAS-containing products. Because PFAS and their terminal breakdown products are widely used, chemically stable, persistent in the environment, and because some have a long elimination half-life, PFAS have been described as ‘forever chemicals’ (Kwiatkowski et al., 2020). These compounding factors have resulted in ubiquitous detection of some PFAS in human and wildlife blood and tissue samples at a global level, with some compounds displaying significant potential for bioaccumulation in wildlife, at up to 100 times the concentrations seen in the environment, making them a global environmental and public health concern (De Silva et al., 2016; Bjerregaard-Olesen et al., 2017; Liu et al., 2018; Abercrombie et al., 2019; Sunderland et al., 2019; Guillette et al., 2020).

### PFAS Exposure in Humans

Human exposure to PFAS may occur through a combination of ingestion, inhalation, and dermal contact (DeLuca et al., 2021). Dietary intake of contaminated food and water represents the largest source of exposure for most adults (Sunderland et al., 2019). However, the relative contribution of exposure routes varies across demographic groups and populations. Furthermore, subpopulations are likely to experience higher PFAS exposure than the general population due to point-source contamination or occupational exposures.

Internationally, PFAS have been found to be ubiquitous in human blood. In a literature review of 87 papers reporting PFAS blood concentrations across different populations globally, Jian et al. (2018) found median concentrations of PFAS in human serum ranging between 0.01 and 10,400 ng/ml. The highest reported concentrations were found in fishery employees in China, likely resulting from increased dietary consumption of contaminated fish (Jian et al., 2018). Piekarski et al. (2020) analyzed thirty-five studies and estimated that global concentrations of perfluoroalkyl acids (PFAAs) in adult serum of the general population (i.e. those without an identified point-source of contamination or occupational exposure) ranged from 0.5–35.5 ng/ml, while the range for humans with known occupational exposure or drinking water contamination was estimated to be between 12.7 and 2,190 ng/ml (Piekarski et al., 2020). Those findings were supported by another review, reporting that serum concentrations of PFAS in occupationally exposed individuals were 1–4 orders of magnitude higher than the general population (De Silva et al., 2021).

Maternal and infant exposure to PFAS has also been reported at a global scale, with studies reporting PFAS detection in populations from Asia, Europe, North America, Africa, and Oceania (Liu et al., 2020). Identification of PFAS in maternal serum and breastmilk highlights placental transfer and lactation as important routes of pre- and perinatal exposures during critical periods of development. The placenta serves as an important biological barrier with the ability to allocate nutrients, hormones, and growth factors to the fetus while also limiting fetal exposure to some toxic substances. PFAS have been detected in serum from maternal, cord, and newborn blood demonstrating that these chemicals are capable of passing through the placental barrier, with estimates of the efficiency of placental transfer ranging from 30—79% (Gützkow et al., 2012; Ma et al., 2021). In addition, breast milk has been shown to account for 83—99% of PFAS total daily intake for infants (Haug et al., 2009; Sundström et al., 2011; Gützkow et al., 2012; Winkens et al., 2017; Liu et al., 2020; Ma et al., 2021). Beyond direct maternal transfer, infants and toddlers may experience higher exposures than adults for several additional reasons. These include reduced functional capacity of biological barriers and metabolic enzymes responsible for xenobiotic detoxification, higher respiration rates and food consumption relative to body weight, and behaviors that increase their contact with contaminated media, such as crawling and frequent hand-to-mouth behaviors. Specifically, infants that are fed formula may experience some of the highest daily exposure levels relative to body weight, as powder formula is mixed with water (Goeden 2018; Goeden et al., 2019; Blake and Fenton 2020). Collectively, infants and toddlers show higher estimated daily intake, relative to body weight, compared to adults reaching peak serum concentrations before 2 years of age (Winkens et al., 2017).

### PFAS Toxicity and Regulation

Perfluorooctanoic acid (PFOA) and perfluorooctanesulfonic acid (PFOS) are the most commonly detected perfluorinated compounds in biological samples, with detectable levels of PFOS reported by the U.S. Center for Disease Control and Prevention (CDC) in the blood of 98% of a representative population of Americans (Sunderland et al., 2019). As a reflection of their ubiquitous and frequent detection, toxicological and exposure data on PFOS and PFOA is abundant in the literature. Numerous studies have demonstrated toxicity in a variety of domains, including hepatotoxicity, nephrotoxicity, developmental toxicity, immunotoxicity, and endocrine toxicity, which has led to further scrutiny of this chemical class as a whole (Lau et al., 2004, 2007; Cousins et al., 2020; Kwiatkowski et al., 2020).

In 2009, PFOS and its salts, and perfluorooctane sulfonyl fluoride (PFOSF), were listed as Persistent Organic Pollutants (POPs) in Annex B of the Stockholm convention, an international treaty written by the United Nations to protect human and environmental health from POPs (UNEP, 2009). A decade later, in 2019, PFOA and its salts were added to Annex A of the Stockholm convention (UNEP, 2019). Those listings designate PFOS and PFOA as chemicals of global concern for human and environmental health, and consequently as priorities for regulation through restriction and elimination of production and use. In the United States, a country which is not a signatory of the Stockholm Convention, the 2010/2015 PFOA Stewardship Program, implemented by the US Environmental Protection Agency (EPA), led to the elimination of the use of PFOA and other long-chain PFAS by several major companies in the PFAS manufacturing industry (EPA, 2022).

For the purposes of this review, PFAS containing seven or more carbons will be considered “long-chain” compounds, and PFAS containing six carbons or less are considered “short-chain” compounds, as they have been defined previously (Conder et al., 2008; Cousins et al., 2020). Phase-out efforts of some PFAS, focused on elimination of a small subset of long-chain compounds including PFOS and PFOA, has spurred global trends toward a decreased abundance for those compounds (Kato et al., 2011; Seo et al., 2018). However, since efforts to limit production of these compounds were initiated, there has been a concurrent increase in production of short-chain PFAS (Kato et al., 2011; Sunderland et al., 2019). Human and wildlife exposure to short-chain PFAS congeners, as well as their toxicity and environmental fate, is not well understood (Sunderland et al., 2019).

When assessing the hazards of PFAS, bioaccumulative potential is a critical parameter to consider, as this governs absorption and distribution of PFAS throughout the body. Carbon chain length has been proposed to be the most important factor in determining the bioaccumulative nature of PFAS, leading some researchers to suggest that shorter chain alternatives to PFOS and PFOA are inherently safer (Renner, 2006; Olsen et al., 2007; Conder et al., 2008). However, recent evidence suggests that some of the short-chain alternatives may be equally persistent and bioaccumulative as PFOS and PFOA (Wilkinson et al., 2017; Gomis et al., 2018; Ateia et al., 2019). In fact, research conducted by Shi et al. (2018) suggests that one factor associated with increased bioaccumulation in long-chain PFAS, hydrophobicity, may have the opposite effect on bioaccumulative potential in short-chain PFAS. However, the literature is disproportionately lacking in research on the bioaccumulative potential, fate and environmental transport, and toxicity of short-chain PFAS, and additional exposure and hazards characterization is needed to understand the relative risk of short-chain PFAS (Ateia et al., 2019).

Due to the vast structural diversity of PFAS, consisting of thousands of different compounds, comprehensively assessing their exposure hazards and risks on an individual, compound by compound basis, for regulatory purposes, would be excessively time-consuming and expensive (Kwiatkowski et al., 2020). Therefore, assessing the parameters important for toxic outcomes in PFAS based upon chemical class, as described previously by Cousins et al. (2020) and Kwiatkowski et al. (2020) is a more effective and realistic approach that is essential for minimizing the hazards of PFAS exposure.

### Nervous System Susceptibility to Toxicant Exposure

The US EPA has defined neurotoxicity as an adverse change in structure and/or function of the central or peripheral nervous system measured at the neurochemical, behavioral, neurophysiological, or anatomical level (Tilson et al., 1995; Tamm and Ceccatelli, 2017). Classically, exposure to well-characterized neurotoxicants, such as lead, mercury, and organophosphate pesticides, culminates in neuronal cell death and other quantifiable neural pathologies. However, we recognize that some contaminants, like endocrine disrupting chemicals, do not fit the classical definition of neurotoxicant, but have profound and biologically relevant impacts on neurophysiology and behavior (Diamanti-Kandarakis et al., 2009; Zoeller et al., 2012; Patisaul and Belcher, 2017). For the purposes of this review, “neurotoxicity” will be used as an inclusive term referencing the impact of PFAS on neurophysiology and behaviors, regardless of mechanism.

Whereas the toxicity of some PFAS to many organ systems has been explored, studies on the neurotoxicity of PFAS are underrepresented in the literature (Piekarski et al., 2020). As the brain is most susceptible to penetration by lipophilic compounds, there is cause for concern that some PFAS may elicit considerable neurotoxic effects. In addition, the blood is one of the major compartments in which PFAS partitions, due to the relatively low affinity binding of PFAS to serum albumin (Maestri et al., 2006; Kudo et al., 2007; Bogdanska et al., 2011; Jackson et al., 2021; Robuck et al., 2021). Thus, due to the highly vascularized nature of the brain, there is enhanced risk of brain exposure to toxicants found in the blood, including PFAS. Finally, the brain contains a high density of non-renewable and long-lived neuronal cells, which can persist throughout life (Esiri, 2007). Therefore, toxic insults that cause damage to neurons, especially during development, may have long-lasting adverse consequences.

#### The Influence of the Blood Brain Barrier on Toxicant Susceptibility

The brain is protected from direct exposure to many (especially ionized) compounds in the blood by the blood brain barrier (BBB). The BBB is a semi-permeable endothelial cell lining that acts as a boundary between blood and brain tissue, maintaining chemical homeostasis by regulating the flow of chemicals into and out of the brain, through metabolism and mechanisms of active and passive transport (Banks, 2009; Cipolla, 2009). Xenobiotic metabolism serves as a protective mechanism at the BBB as endothelial cells, pericytes, and glia express numerous metabolic enzymes including cytochrome P450s (Dauchy et al., 2008, 2009). The BBB is characterized by very low rates of paracellular and transcellular molecular transport, achieved through continuous intercellular tight junctions and a lack of endothelial cell fenestrations (Obermeier et al., 2013). The adult BBB is remarkably effective in providing nutrients and oxygen to the brain through nutrient transporters, while protecting the brain from many toxic substances through efflux transporters (Obermeier et al., 2013).

**FIGURE 2:**
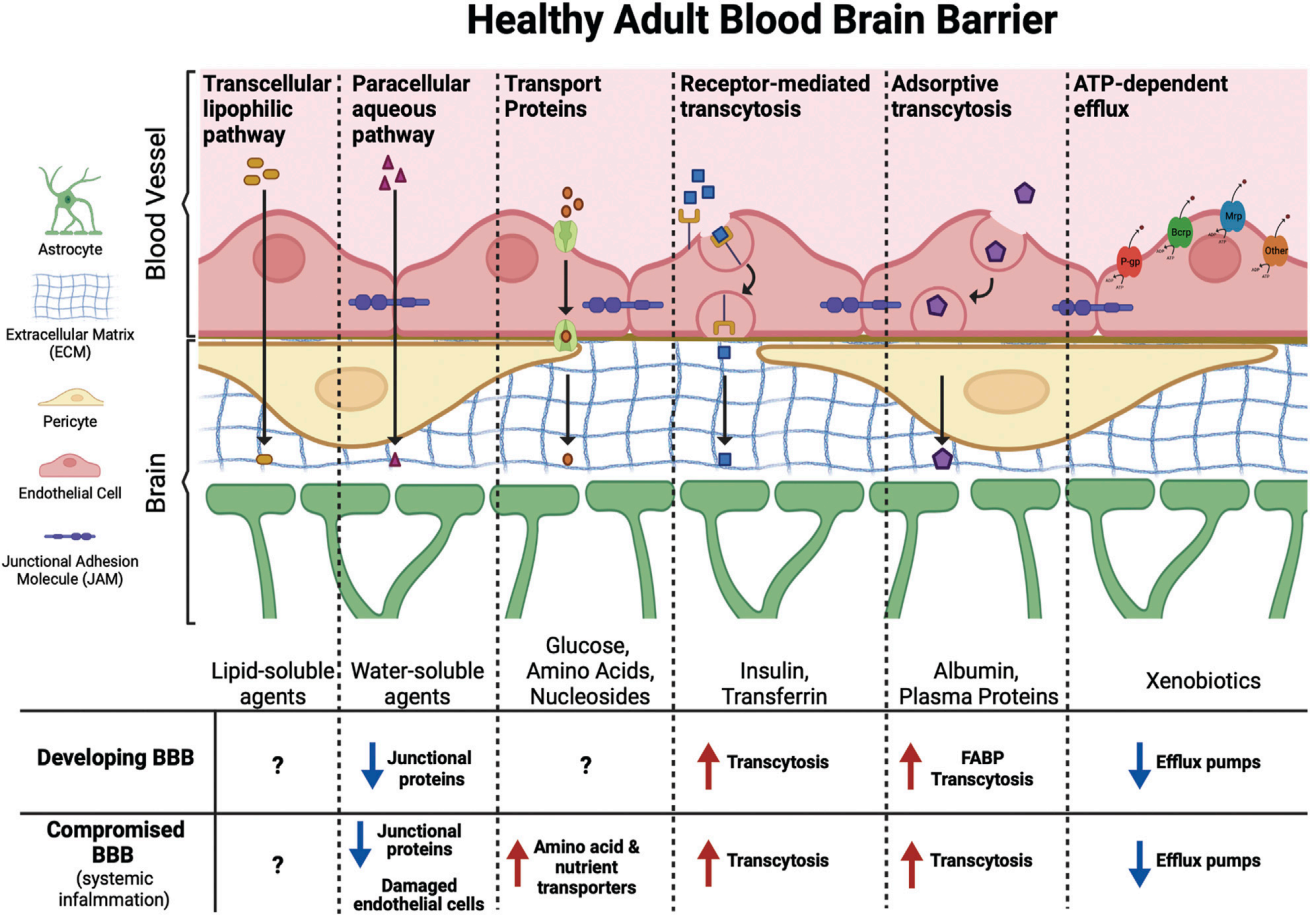
Molecules of different sizes and biochemical properties can gain access to the brain *via* diverse mechanisms. This includes facilitated (transport proteins, receptor-mediated transcytosis, and adsorptive transcytosis) and passive (transcellular lipophilic and paracellular aqueous pathways) transport across the BBB. ATP-dependent efflux transporters protect the brain from toxic xenobiotics and endogenous metabolites. These transporters include p-glycoprotein (P-gp), breast cancer resistance protein (Brcp), and multidrug resistance-associated protein (Mrp) among others. The developing BBB has not yet reached its full functional capacity, with fewer junctional proteins, increased transcytosis, and lower expression of efflux pumps compared to the adult BBB may leave the fetal and infant brain more vulnerable to PFAS exposure. Similarly, certain ailments, such as conditions that cause systemic inflammation, can compromise the BBB leading to increased transport of xenobiotics into the brain.

As reviewed by Banks (2009), two of the major mechanisms by which compounds may cross the BBB are transmembrane diffusion and saturable active transport systems (Figure 2). Transmembrane diffusion is the route typically taken by smaller molecules (<500 Da). Unbound or free fractions of lipophilic compounds can generally diffuse freely across the BBB, however it is important to recognize that diffusion from circulation to the CNS is impacted by relative charge, affinity for both serum and intracellular proteins, and three-dimensional chemical structure (Banks, 2009). Compounds of high molecular weight, or those containing charged components, are more likely to cross the BBB through saturable transport systems (Banks, 2009). These saturable transport systems operate *via* ligand binding to receptors or transporters, *via* ATP-dependent or independent mechanisms, facilitating the influx of compounds at approximately ten times the rate of diffusion (Banks, 2009).

**FIGURE 3:**
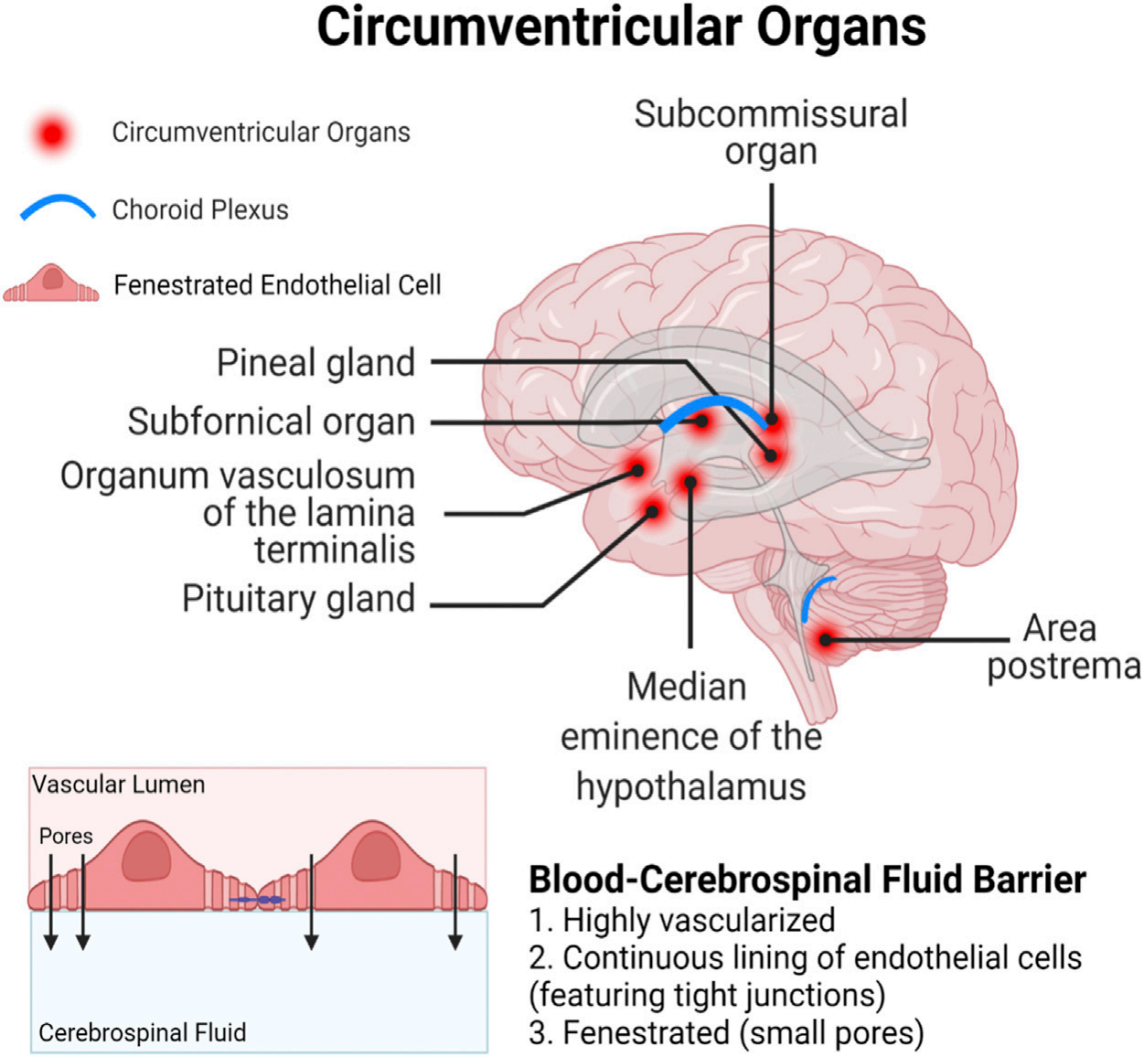
Circumventricular organs represent a particularly vulnerable site for xenobiotic entry, as they are in close proximity to the blood-cerebrospinal fluid barrier which is lined with fenestrated (porous) endothelial cells.

Both long-chain and short-chain PFAS are able competitively bind proteins, primarily serum albumin and fatty acid binding proteins (FABPs), that result in circulatory transport and intracellular uptake into tissues (Jackson et al., 2021; Luebker et al., 2002; Sheng et al., 2018; Zhang et al., 2013). Serum albumin is the predominant PFAS binding protein in the blood, and PFAS binding to albumin is a critical mediator of PFAS distribution in different organ systems (Jackson et al., 2021). FABPs are intracellular proteins expressed widely throughout the body in many different tissue types. Three FABP subtypes are expressed in the brain and play important roles in uptake and metabolism of fatty acids in processes of brain development and neuronal regeneration; the *FABP3* gene encodes heart FABP (H-FABP), *FABP5* encodes epidermal FABP (E-FABP), and *FABP7* encodes brain FABP (B-FABP) (Owada, 2008). Although studies have only demonstrated PFAS binding to liver FABPs (L-FABPs; FABP1), it is likely that FABPs may also play a role in PFAS partitioning within the CNS. Finally, compounds could bypass the BBB entirely and enter the brain through one of the circumventricular organs, which lack a fully functional BBB (Figure 3; Ganong, 2000). The circumventricular organs are small, highly permeable organs in close proximity to the brain’s third and fourth ventricles, which utilize fenestrated capillaries to allow hormones to leave the brain without crossing the BBB, and similarly allow other substances to enter the brain (Ganong, 2000).

#### BBB in the Developing Brain

The developing brain is more susceptible to damage from toxicant exposure than the adult brain, in part due to the intricate and coordinated signaling events that take place as the rudimentary structures of the central nervous system (CNS) are formed and organized. These signaling events regulate complex processes such as proliferation, differentiation, migration, apoptosis, and synaptic pruning, making the developing brain highly responsive to intrinsic and extrinsic stimuli. Therefore, exposure to contaminants during this window can result in unwanted long-term changes in shape, size, and functionality of the brain (Heyer and Meredith, 2017).

Although the adult brain is capable of limited neurogenesis, the developing brain experiences a period of rapid neurogenesis in which an estimated 4.6 million neurons are generated every hour between birth and 1.5 years of age in humans (Eriksson et al., 1998; Silbereis et al., 2016). Therefore, toxicant-induced damage to the brain, especially during these critical windows of development, can have long-lasting impacts throughout an individual’s life (Heyer and Meredith, 2017). Although there are well-described differences in permeability to toxicants in the developing BBB as opposed to the adult BBB, the differential mechanisms by which chemicals cross the barriers at different developmental timepoints are not well-defined. However, rates of transcytosis, or vesicular trafficking between blood-facing (luminal) and brain-facing (abluminal) endothelial cells, are still relatively high during early embryonic development, and the BBB is not yet at its full functional capacity. For example, the embryonic BBB exhibits decreased expression of P-glycoprotein (P-gp) efflux transporters, the primary ATP-binding cassette (ABC) transporter responsible for xenobiotic clearance at the BBB, and these lower levels of efflux activities can lead to increased xenobiotic concentrations in the brain (Ek et al., 2010; Petropoulos et al., 2010; Obermeier et al., 2013; Chow and Gu, 2017; Langen et al., 2019). Additionally, two of the FABPs expressed in the brain, E-FABPs and B-FABPs, have greater expression during embryonic development and only weak expression in the brain in adulthood, offering a potentially unique avenue for increased intracellular sequestration of PFAS in the developing brain (Owada, 2008). Research also suggests that selective transport of plasma proteins and small molecules across the epithelial layer of the choroid plexus is an uptake mechanism unique to the developing brain, however it is unclear whether this mechanism would allow the crossing of PFAS-bound proteins into the cerebrospinal fluid (CSF; Ek et al., 2003, Ek et al., 2012; Johansson et al., 2006).

### PFAS and Neurotoxicity

The literature, predominantly focused on PFOA and PFOS, provides evidence demonstrating that PFAS can elicit neurotoxic effects, with consequences at the neurobehavioral, neurophysiological, and neurochemical levels (Mariussen, 2012; Wang Y. et al., 2019; Foguth R. et al., 2020; Piekarski et al., 2020; Cao and Ng, 2021). In addition to PFAS’ ability to cross the BBB, some are able to disrupt the functional integrity of the barrier by disrupting endothelial tight junctions and efflux transporter activities, associated with increases in oxidative stress (Wang J. et al., 2018). Although the BBB has the ability to metabolize xenobiotics, fully fluorinated PFAAs are recalcitrant to metabolism and biotransformation (Kudo and Kawashima, 2003; Lau et al., 2007).

While the literature suggests that PFAS may act as neurotoxicants, inconsistency across study designs and lack of experimental relevance to human and wildlife exposures poses challenges in interpretation of experimental findings (Piekarski et al., 2020). The goal of the current review is to outline the available evidence of neurotoxicity of a wide range of PFAS, broken into the general categories of long- and short-chain compounds. Potential neurotoxicity will be evaluated as it pertains to neurobehavioral endpoints, focusing on probable chemical and molecular mechanistic explanations for observed adverse impacts. We will focus on assessment of the neurotoxic potential during sensitive exposure windows and in sensitive populations, both of which may exacerbate the neurotoxic effects of the chemicals. Finally, we will highlight the gaps in the current research, and suggest areas of research focus that should be prioritized in future studies.

## PFAS Uptake and Accumulation in the Brain

While PFAS are most concentrated in protein rich tissues of the body, primarily the liver and serum, they have also been detected in nervous tissues of humans, experimentally exposed laboratory animals, and diverse taxa of environmentally exposed wildlife. In addition to humans, PFAS have been detected in the brains of numerous organisms, including mice, rats, frogs, fish, birds, and marine and arctic mammals (Ahrens et al., 2009; Bogdanska et al., 2011; Rubarth et al., 2011; Shi et al., 2012; Greaves et al., 2013; Iwabuchi et al., 2017; Foguth R. M. et al., 2020). The concentrations of PFAS in the brain vary appreciably depending on duration and route of exposure, however, for the most well studied PFAS (PFOA and PFOS), accumulation in the brain is proportional to exposure dose (Austin et al., 2003; Kudo et al., 2007; Cui et al., 2009; Cao and Ng, 2021).

**FIGURE 4:**
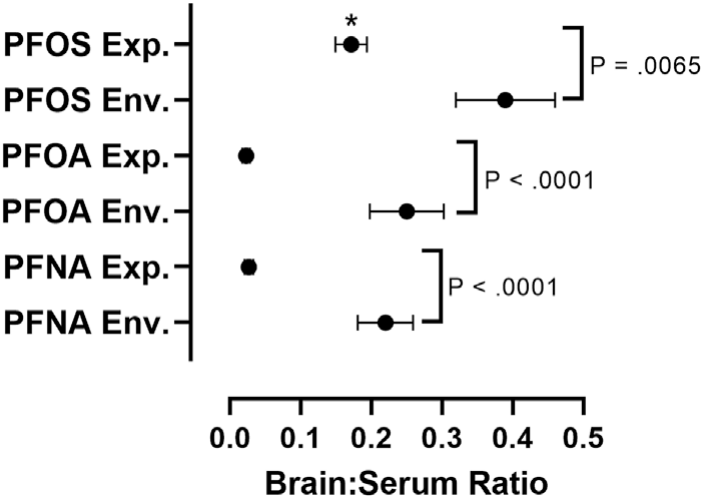
Comparative assessment of average brain:serum ratios of long-chain PFAAs in experimental animal exposure studies vs. environmental exposure studies. Mean ratios represented by black circles with error bars representing ± standard error of the mean. A two-way analysis of variance (ANOVA) on log-transformed brain:serum PFAS ratios with long chain PFAA congener (PFOS, PFOA, PFNA) and exposure type (experimental animal exposure vs environmental exposure) revealed main effects of PFAA congener, F (2, 85) = 26.47, *p* < 0.0001, and exposure type, F (1, 85) = 114.7, *p* < 0.0001. A Šidák multiple comparisons post-hoc analysis with α = 0.05 indicated that environmental brain:serum ratios were greater than experimental brain:serum ratios for PFOS (*p* = 0.0065), PFOA (*p* < 0.0001), and PFNA (*p* < 0.0001). Asterisks indicate that experimental brain:serum ratios of PFOS were greater than PFOA and PFNA (*p* < 0.0001). Five outliers were removed, identified by the Rout method (1 experimental PFOS, three experimental PFOA, one experimental PFNA). Sample sizes varied across groups: PFOS experimental (*n* = 33), PFOS environmental (*n* = 11), PFOA experimental (*n* = 15), PFOA environmental (*n* = 11), PFNA experimental (*n* = 11), PFNA environmental (*n* = 10). Details on exposure studies can be found in Supplementary Tables S2, S3.

In order to assess differences in PFAS accumulation in the brain in *in vivo* experimental studies versus environmental exposure assessments, we calculated brain:serum ratios of individual PFAS congeners from 13 different experimental animal studies and eight environmental exposure assessments (Figure 4; Supplementary Tables S2, S3). To find values used in our meta-analysis, a literature review was conducted using combinations of the search terms “PFAS,” “Brain,” “Serum,” “Blood,” “Tissue Distribution” and “Concentrations,” utilizing the search engines Google Scholar and NCBI PubMed, Studies cited within papers found using these search terms were also utilized in our analysis. Studies reporting raw values for concentrations of PFOA, PFOS, or PFNA in the brain and serum (or blood/plasma), or a ratio of brain:serum PFAS levels, were included in statistical analysis. Data was extracted from the main body of the text or the supplementary materials of the referenced studies. Brain:serum ratio data for PFOA, PFOS, and PFNA in both exposure categories fit a lognormal distribution, and therefore log-transformed values were used in statistical analysis. Five outliers were removed (1 experimental PFOS, three experimental PFOA, one experimental PFNA), identified by the Robust Regression and Outlier Removal (ROUT) method (Supplementary Table S2). A two-way analysis of variance (ANOVA) with long chain PFAA congener (PFOS, PFOA, PFNA) and exposure type (experimental animal exposure vs environmental exposure) revealed main effects of PFAA congener, F (2, 85) = 26.47, *p* < 0.0001, and exposure type, F (1, 85) = 114.7, *p* < 0.0001. A Šidák multiple comparisons post-hoc analysis with α = 0.05 indicated that environmental brain:serum ratios were greater than experimental brain:serum ratios for PFOS (*p* = 0.0065), PFOA (*p* < 0.0001), and PFNA (*p* < 0.0001). Additionally, experimental brain:serum ratios of PFOS were significantly greater than PFOA and PFNA (*p* < 0.0001). Sample sizes varied across groups: PFOS experimental (*n* = 33), PFOS environmental (*n* = 11), PFOA experimental (*n* = 15), PFOA environmental (*n* = 11), PFNA experimental (*n* = 11), PFNA environmental (*n* = 10).

The brain to serum ratio of long-chain PFAA concentrations, particularly PFOS, PFOA, and PFNA, are significantly greater in animals exposed environmentally than in experimentally exposed laboratory animals (Figure 4; Supplementary Tables S2, S3). This difference is likely the result of differences in exposure concentrations and duration. In general, most environmental exposure assessments are made from organisms following chronic exposure to lower concentrations of multiple PFAS, as opposed to the high dose shorter-term exposures that dominate the experimental literature (Piekarski et al., 2020). Additionally, brain:serum ratios of PFOS were significantly greater than PFOA and PFNA in experimental studies (Figure 4). This increased brain:serum ratio of PFOS highlights the enhanced uptake of 8-carbon sulfonated PFAAs into the brain, in comparison to 8- and 9-carbon carboxylated compounds (Figure 4).

Available primary data for statistical meta-analysis was insufficient for assessing brain:serum ratios for other PFAS compounds, however the data suggests a similar trend toward increased brain partitioning after environmental exposures (Supplementary Tables S2, S3). Possible differences in accumulation of PFAS in the brains from experimental vs environmental exposures could result from experimental bias, as the majority of experimental studies utilize PFOS and PFOA, skewing the data towards brain:serum distribution ratios of 8-carbon compounds. This possibility is supported in a study by Wen et al. (2019) that found that in zebrafish (*Danio rerio*), brain:serum ratios for a subset of structurally diverse PFAA congeners markedly differed, with the proportion of PFAS in brain increasing as the perfluoroalkyl chain length increased. Additionally, studies measuring concentrations of different PFAS in environmentally exposed harbor seals (*Phoca vitulina*) and red-throated divers (*Gavia stellata*) found that the long-chain PFAS PFDoA, perfluoroundecanoic acid (PFUnDA), perfluorotridecanoic acid (PFTriDA), perfluorotetradecanoic acid (PFTeDA), and perfluorooctane sulfonamide (PFOSA) partitioning to brain was similar to other body compartments (Ahrens et al., 2009; Rubarth et al., 2011). It is important to note that in environmentally exposed animals, differences in PFAS accumulation across compounds may be influenced by a number of uncontrolled variables, including differences in exposure levels to different PFAS, and species or sex-specific differences in elimination rates for each compound. Finally, there is also additional experimental bias across available biomonitoring and experimental studies because of variations in the numbers of different compounds tested, exposure routes, durations of exposures, and comparisons across a variety of different species and populations.

The concentrations of different PFAS congeners found in the serum, and those that sequester to the CNS, vary based on the chemical structure of the compound. One epidemiologic study determined that there is considerable variation in BBB penetration for different PFAS compounds by calculating a penetration ratio (R_PFAS_) for each congener detected in CSF and serum (R_PFAS_ = PFAS_CSF_/PFAS_serum_; Wang J. et al., 2018). Furthermore, Wang J. et al. (2018) determined that the integrity of the blood brain barrier (BBB), as measured by a commonly used parameter R_alb_ (the ratio of albumin in the CSF:serum), was more important than any other clinical parameter in determining the extent to which PFAS in the blood can enter the brain. Integrity of the BBB can be compromised because of exposures to certain toxicants, such as pesticides, mixed vehicle emissions, and tobacco smoke, as well as certain health conditions including diabetes and neurological disorders reviewed in *Neurotoxicity of PFAS in Sensitive Populations*, through molecular alterations in barrier function (Gupta et al., 1999; Weiss et al., 2009; Mazzone et al., 2010; Oppenheim et al., 2013; Prasad et al., 2014, 2015; Erdő et al., 2017; Suwannasual et al., 2018; Martinez and Al-Ahmad, 2019). PFAS can disrupt BBB integrity by various mechanisms including disruption of endothelial tight junctions, actin filament remodeling induced by oxidative stress, and decreased activity of efflux transporters (Qian et al., 2010; Wang X. et al., 2011; Reistad et al., 2013; Cannon et al., 2020). Therefore, PFAS exposure may impact access and accumulation of contaminants in the brain, by increasing permeability at the BBB.

Endothelial tight junctions also play a primary role in the physical regulation of molecular transport at the BBB, and working in conjunction with astrocytes, protect the brain from toxic xenobiotics (Figure 2; Abbott, 2002). PFAS are capable of both directly and indirectly disrupting endothelial tight junction structure and function. One *in vitro* study has shown that exposure to 50 µM concentrations of PFOS caused disassembly of endothelial tight junctions in human brain microvascular endothelial cells, in part through the disruption of membrane proteins occludin and claudin-5, leading to increased BBB permeability (Wang X. et al., 2011). Further, PFOS was found to decrease expression of endothelial tight junction proteins after *in vivo* exposure in outbred ICR mice, at 0.25 mg/kg/day for claudin-11, and at very high doses for occludin and claudin-5 (25 mg/kg/day), and ZO-1 (50 mg/kg/day) (Yu et al., 2020). Those changes in endothelial tight junction proteins led to astrocytic damage at the BBB, again resulting in higher concentrations of PFOS in the brain. Another mechanism by which PFAS disrupt the endothelial barrier of the BBB is through generation of reactive oxygen species (ROS), that are ultimately able to induce remodeling of actin filaments (Qian et al., 2010). In cultured human microvascular epithelial cells (HMVEC), Qian et al. (2010) demonstrated that exposure to PFOS at concentrations as low as 2 µM induced generation of ROS, which increased the permeability of an HMVEC monolayer *via* remodeling of actin filaments. Actin filaments are a fundamental component of the cytoskeleton and play an important role in maintaining the structural and functional integrity of endothelial tight junctions at the BBB (Lai et al., 2005). Whereas this actin filament remodeling has only been demonstrated in response to PFOS exposure, several other *in vitro* studies have also demonstrated the ability of PFOS, PFOA, and PFOSA, to generate ROS in neurons and astrocytes (Lee et al., 2012; Reistad et al., 2013; Chen et al., 2014; Li Z. et al., 2017).

Although lipophilicity is important for transmembrane diffusion through the BBB, some compounds that are too lipophilic can be discharged after crossing into the brain by the P-gp efflux transporter (Abbott, 2002; Banks, 2009). Cannon et al. (2020) isolated rat brain capillaries to assess the effects of Perfluoro (2-methyl-3-oxahexanoic) acid (GenX), a perfluoroether carboxylic acid that was introduced as an alternative for PFOA and PFOS, on the BBB. This study found that GenX decreases the transport activity of P-gp and breast cancer resistance protein (BCRP), two ABC transporters which actively limit endogenous ligands, xenobiotics, and drugs from reaching sensitive target tissues, including the brain. Thus, disruption of P-gp and BCRP by GenX could cause a positive feedback loop by which exposure to PFAS decreases the capacity for efflux of xenobiotics, including PFAS, from the brain. Discontinuation of exposure to GenX allowed P-gp transport activity to return to baseline. However, the decreased transport activity of BCRP following GenX exposure was not reversible, suggesting that GenX may permanently alter BBB xenobiotic efflux transport following short-term exposure *in vitro* (Cannon et al., 2020).

### Brain Region Specific Accumulation

Due to the diversity of PFAS, perfluorinated compounds with varying chemical structures are likely to cross the BBB through multiple mechanisms, which might result in different patterns of distribution in the CNS (Greaves et al., 2013; Cao and Ng, 2021). Numerous studies have analyzed the distribution of PFAS in different brain structures with some finding that concentrations of individual PFAS congeners varied across brain region. The brain stem, hypothalamus, and thalamus are some of the most highly perfused and lipid-rich brain regions, while the frontal and temporal cortex are characterized as having lower lipid content and relatively low perfusion (Cipolla, 2009; Greaves et al., 2013). Two studies analyzing the concentrations of different PFAS in brains of polar bear (*Ursus maritimus*) collected after annual Inuit subsistence hunting in East Greenland, reported PFAS concentrations of several different carboxylated and sulfonated congeners across different brain regions (Greaves et al., 2013; Eggers Pedersen et al., 2015). The highest concentrations of PFAS were reported in the hypothalamus, brain stem, thalamus, and cerebellum, whereas the lowest concentrations were observed in the cortex (Greaves et al., 2013; Eggers Pedersen et al., 2015). Greaves et al. (2013) also reported a positive correlation between longer chain perfluorinated carboxylic acids (PFCAs) and extractable lipid content in the brainstem and cerebellum, hypothesizing that PFCAs with carbon chain lengths between 10 and 15 may be binding to serum proteins and crossing the BBB in a similar way to saturated fatty acids. This hypothesis is supported by *in vitro* work demonstrating that proteins like human serum albumin and human L-FABP have optimal affinity for PFAS with carbon chain length around 10 (Zhang et al., 2013; Jackson et al., 2021).

Studies in a rodent model and human brain samples showed a similar pattern of distribution in the brain for PFOS, perfluorohexanesulfonic acid (PFHxS), and perfluorohexanoic acid (PFHxA) as was seen in the polar bears. Although statistical comparisons of PFAS concentrations in different brain regions were not performed, both studies reported brain-region-specific accumulation, demonstrating increased exposure in the hypothalamus and decreased exposure in the cortex (Austin et al., 2003; Di Nisio et al., 2022). Austin et al. (2003) reported the highest concentration of PFOS in the hypothalamus (15,706 ng/g), and the lowest concentration in the cortex (4,487 ng/g), after exposing female rats to 10 mg/kg/day *via* intraperitoneal injection for 2 weeks. Similarly, Di Nisio et al. (2022) analyzed PFOA, PFHxS, and PFHxA concentrations across brain regions in 5 deceased human male subjects and found the greatest concentrations in the hypothalamus across all compounds (206.93 ± 78.23 ng/g). The brain region with the lowest concentrations for each individual compounds varied, but the lowest overall PFAS concentrations were found in the thalamus (19.75 ± 6.73 ng/g), midbrain (24.01 ± 6.65 ng/g), and frontal lobe (29.58 ± 10.73 ng/g). These relatively increased concentrations seen in the hypothalamus may be partially attributed to the presence of the median eminence, a circumventricular organ that secretes hormones produced in the brain (Figure 3). Therefore, toxic compounds may gain access to the hypothalamus without needing to cross the BBB (Ganong, 2000). Collectively, environmental and experimental evidence highlight the hypothalamus as a brain region with disproportionately high accumulation of PFAS.

## Neurotoxic Effects of Adult Exposure to PFAS

### Long-Chain PFAS

Neurobehavioral impacts, such as impaired spatial learning and memory, after exposure to long-chain PFAS in adult animal models have been reported, though exposure levels tended to be high and findings were inconsistent across studies. Although not as prevalent or consistent in the literature, experimental evidence of PFAS-induced impacts on anxiety-like behavior, motor activity, and coordination have also been reported.

Exposure to PFOS has been associated with deficits in spatial learning and memory in a Morris Water Maze task in adult mice (Fuentes et al., 2007b; Long et al., 2013). Fuentes et al. (2007b) exposed male mice to 3 or 6 mg/kg/day of PFOS for 4 weeks and found that both PFOS treated groups exhibited decreased spatial memory retention compared to their control counterparts in a Morris Water Maze task, with this deficit persisting for 4 weeks after exposure. In another study with male and female mice, Long et al. (2013) exposed animals to 0.43, 2.15, or 10.75 mg/kg PFOS daily for 3 months, and also noted a dose-dependent impairment in spatial and learning memory. Similarly, in a novel object recognition test conducted by Kawabata et al. (2017), adult male rats exposed to a single oral dose of 50 mg/kg perfluorododecanoic acid (PFDoA) experienced decreased memory and attention in a dose dependent fashion. However, rats exposed to the same dosage of PFOA or perfluorodecanoic acid (PFDA) did not exhibit memory deficits in the novel object recognition task, and animals in all treatment groups exhibited intact working memory (Kawabata et al., 2017). In a less traditional model of learning and memory, another study utilized an associative learning assay in nematodes (*Caenorhabditis elegans*) to assess the role PFOS plays in altering chemotaxis behavior, finding that 20 μM PFOS in culture medium reduced learning ability (Chen et al., 2014). Furthermore, PFOS exposure caused down-regulation of *gcy-5*, a gene that encodes chemoreceptors in amphidial chemosensory (ASE) neurons, which are essential for the assayed chemotaxis behavior (Chen et al., 2014). These studies highlight that the neural circuits and brain regions that regulate learning and memory are vulnerable to PFOS.

Fuentes et al. (2007b) also noted that deficits in spatial memory were accompanied by a subtle increase in anxiety-like behavior for both PFOS exposed groups in an open field test, with exposed mice displaying decreased rearing behavior in the higher dose group and decreased time spent in the middle of the open field in the lower dose group. These findings were not observed throughout the entire 15-min test but were transient, with decreases in rearing and time in center observed during the 0–5 min and 5–10 min timepoints, respectively (Fuentes et al., 2007b). While subtle, the transient nature of the anxiety-like behaviors reported are biologically relevant in the context of the open field task, as animal behavior changes with experience and habituation during long testing paradigms. Furthermore, the beginning of the open field task, particularly the first 5 min, is considered a novel experience for the animal and provides the most salient information about anxiety-related behaviors. Therefore, the observed reduction in exploratory behavior early in the open field task indicates that behavioral responses to a novel environment are altered in PFOS exposed animals. In contrast, rats exposed to PFDoA showed evidence of decreased anxiety, by spending more time than control rats in the open arm of an elevated plus maze, and exhibited no changes in anxiety-related behavior when tested in the open field (Kawabata et al., 2017). While these studies both utilize oral gavage as their method of dosing, the amount and duration of exposure is substantially different, wherein Fuentes et al. (2007b) used a 4-weeks lower dose (3 or 6 mg/kg) paradigm and Kawabata et al. (2017) exposed their animals to a single high dose (50 mg/kg). Furthermore, differences in animal models (*M. musculus* vs. *R. norvegicus*) and the chemicals they were exposed to (PFOS vs. PFDoA) make the lack of consistency in anxiety-like phenotypes unsurprising. Collectively, these studies provide some evidence that the hippocampus, a brain region critical for spatial learning and memory as well as anxiety-like behavior, is vulnerable to long-chain PFAS exposure and warrants further assessment (Bannerman et al., 2004; Sweatt, 2004).

Motor coordination and motor activity were not significantly impacted by exposure to PFOS and PFOA in mice and rats, respectively (Fuentes et al., 2007b; Butenhoff et al., 2012). However, Butenhoff et al. (2012) did observe an abnormal hunched position in all animals receiving 30 mg/kg/day of PFOA for 28 days, with some also exhibiting piloerection and an abnormal gait. In addition, all male animals receiving the highest dose of PFOA exhibited a delayed bilateral pupillary reflex, while histological analysis of the optic nerve showed no apparent changes compared to controls (Butenhoff et al., 2012). Although it’s possible that PFOA-induced neurotoxicity contributes to these phenotypes, it is also important to note that these findings are likely driven by general toxicity and pain response as exposure to 30 mg/kg/day PFOA increased the overall incidence of clinical signs of toxicity in these animals (Butenhoff et al., 2012).

### Short-Chain PFAS

Research detailing the neurotoxicity of short-chain compounds on adult neurobehavior is sparse. To the best of our knowledge, only three *in vivo* studies have been published detailing the neurobehavioral impacts of short chain PFAS compounds on adult animals (Butenhoff et al., 2009a, 2012; Lieder et al., 2009). All three papers were published by the PFAS manufacturer 3M and reported only negative results for the effects of perfluorobutyric acid (PFBA), perfluorobutanesulfonic acid (PFBS), and PFHxS on the nervous system (Butenhoff et al., 2009a, 2012; Lieder et al., 2009). Lieder et al. performed a 90-days oral gavage study in male and female rats, noting no changes in a functional observational battery (FOB), motor activity, or gait in response to 20, 200, or 600 mg/kg PFBS. Unfortunately, detail provided as to how these behavioral assessments were conducted lacked transparency and was inadequate for replication (Lieder et al., 2009). Butenhoff et al. (2012) also reported no changes in FOB or motor activity after exposure to 150 mg/kg/day of PFBA for 28 days *via* oral gavage in rats. The study did report delayed bilateral pupillary reflex, with histological analysis of the optic nerve showing no exposure-related abnormalities (Butenhoff et al., 2012). Similarly, Butenhoff et al. (2009a) reported no changes in FOB, motor activity, or gait in response to daily oral exposure to PFHxS for 21 and 42 days in females and males, respectively. The FOB used in these studies is useful for assessing gross functional deficits, however additional testing paradigms, such as those that evaluate reproductive, cognitive, social, and emotional behaviors, are necessary to gauge the neurotoxic potential of a chemical more completely. While these studies primarily report negative findings, more research is needed utilizing more comprehensive behavioral testing paradigms and additional chemicals before we can make any conclusions about the neurotoxic potential of short-chain PFAS on adult animals, in the context of behavior.

## Neurotoxic Effects of Developmental Exposure to PFAS

Exposures to toxicants during early development are particularly harmful to the brain (Grandjean and Landrigan, 2006). PFAS are no exception to this rule, and it has been well-documented that these chemicals are more toxic to individuals exposed developmentally, and particularly to the developing brain (Lau et al., 2004; Mariussen, 2012; Wang Y. et al., 2019; Piekarski et al., 2020). The brain growth spurt (BGS), a period of rapid growth and development, is a sensitive window that lasts from the third trimester of pregnancy to 2 years of age in humans, while in mice and rats it lasts only through the first 3-4 postnatal weeks (Eriksson, 1997). However, even prior to the BGS, important neurodevelopmental milestones are reached during gestation (Dobbing and Sands, 1979). To encompass the most critical time periods for brain growth and development, the term “developmental exposure” will be defined in this review as the time window beginning at gestational day zero and continuing through the end of the BGS, also referred to as the perinatal window.

More PFAS penetrates to the brain tissue of experimental animals after developmental exposure, in comparison to adult exposures (Chang et al., 2009; Borg et al., 2010; Ishida et al., 2017). Although there is some discrepancy across studies for particular endpoints, the neurobehavioral impacts of PFAS also seem to be more pronounced after developmental exposure (Mariussen, 2012). Animals exposed during the perinatal window exhibited some of the same effects as animals exposed in adulthood, such as disordered learning and memory, and anxiety-like behavior. However, developmentally exposed animals have also demonstrated hyperactivity, disrupted locomotion and habituation, and adverse outcomes in FOB testing.

### Long-Chain PFAS

Across species, the most consistent behavioral finding associated with developmental exposure to long-chain PFAS is impaired motor activity. While studies on PFAS exposure in adult animals have not reported altered motor activity, several compounds have been shown to elicit developmental impairment of motor activity in mice, rats, and zebrafish (Johansson et al., 2008; Butenhoff et al., 2009b; Huang et al., 2010; Wang M. et al., 2011; Chen et al., 2013; Spulber et al., 2014; Hallgren et al., 2015; Goulding et al., 2017; Guo et al., 2018; Reardon et al., 2019; Gaballah et al., 2020; Kim et al., 2020). However, the majority of evidence in rodent models is driven by results for PFOS and PFOA. Male and female Sprague-Dawley (SD) rats exposed to PFOS during the perinatal window, *via* oral administration to dams, displayed hyperactivity and decreased habituation (Butenhoff et al., 2009b; Reardon et al., 2019). The same hyperactivity was exhibited by C57BL/6 mice gestationally exposed to 0.1 mg/kg/day PFOA, from GD seven to weaning (Sobolewski et al., 2014). The observed changes in motor activity may be associated with PFOS/PFOA driven disruption of the developing cholinergic system. In inbred male NMRI mice, a single oral exposure of PFOA or PFOS at PND 10 caused a long-lasting hyperactive phenotype at 2 and 4 months of age, characterized by decreased habituation and altered spontaneous behavior (Johansson et al., 2008; Hallgren et al., 2015). Decreased habituation was observed in the mice after exposure to 8.7 mg/kg PFOA or 11.3 mg/kg PFOS, whereas altered spontaneous behavior was observed after exposure to 0.58 mg/kg PFOA or 0.75 mg/kg PFOS (Johansson et al., 2008; Hallgren et al., 2015). In conjunction with altered spontaneous motor activity, Hallgren et al. (2015) found that PFOS decreased transcription of genes encoding proteins essential to cholinergic system functioning in the brain, including acetylcholinesterase (AchE), nicotinic acetylcholine receptor β2 (nAChR-β2), and muscarinic acetylcholine receptor m5 (mAChR-5). Another study assessing gestational exposure to 1 mg/kg/day PFOA for 17 days in male mice found that PFOA exposure induced a mild increase in locomotor activity at PND 18, compared to control animals (Goulding et al., 2017). Importantly, authors also utilized two drugs, nicotine and methamphetamine hydrochloride, that target nicotinic cholinergic receptors in an agonistic manner, to assess PFOA-induced impacts to the cholinergic system (Goulding et al., 2017). After 17 days of gestational PFOA exposure, male offspring at 6 months of age were subcutaneously injected with a single dose of 80 μg/kg nicotine or vehicle control, and another cohort was intraperitoneally injected with a single dose of 2 mg/kg methamphetamine or vehicle control, and motor activity was examined in an open field test (Goulding et al., 2017). Goulding et al. (2017) did not observe any exposure related effect in response to the subsequent nicotine challenge in adulthood. However, they did report a PFOA-related reduction in hyperactivity after administration of methamphetamine at 6 months of age, compared to control animals (Goulding et al., 2017). In addition to activating nicotinic cholinergic receptors, methamphetamine is a dopaminergic agonist. Thus, authors hypothesized that alterations in methamphetamine-induced motor activity in PFOA-exposed mice, in the absence of nicotine-induced changes, may indicate potential disruption of the dopaminergic system (Goulding et al., 2017).

Current evidence suggests that developmental exposure to PFOA and PFOS increases motor activity in mice and rats and brings attention to the cholinergic system as a potential target and mechanism driving these behavioral changes. However, it is important to remember the limitations and confounding factors of certain behavioral tasks. Tests that assess exploratory and locomotor behavior can be difficult to interpret as aspects of physical activity and emotional response to a novel environment are both at play. For example, Fuentes et al. (2007b) reported that 3-month-old mice gestationally exposed to 6 mg/kg/day PFOS, from GD 12–18, displayed a decrease in distance traveled in an open field. While this may provide seemingly contradictory evidence to the rest of the findings reported here showing hyperactivity, these findings may also be indicative of an anxiety-like response in PFOS exposed animals. For this reason, future studies should employ multiple testing strategies with overlapping behavioral endpoints to better characterize PFAS-associated behavioral phenotypes.

Several studies assessing PFOS exposure in larval zebrafish reported increased swimming speed or hyperactivity in PFOS-exposed adults (Huang et al., 2010; Chen et al., 2013; Gaballah et al., 2020). Another study found that male zebrafish developmentally exposed to 2 µM perfluorononanoic acid (PFNA) experienced hyperactivity, decreased distance traveled, increased aggressive behavior and thigmotaxis, or avoidance of the center of the arena, in adulthood (Jantzen et al., 2016). Jantzen et al. (2016) reported no hyperactivity in fish exposed to 2 µM PFOS or PFOA, although they reported decreased aggression in PFOS-exposed males, and increased anxiety-like behavior in PFOA-exposed females. Notably, multiple studies reported that exposure to PFAS induced bouts of hyperactivity, but decreased overall locomotion and distance traveled (Spulber et al., 2014; Jantzen et al., 2016). In studies reporting exposures in zebrafish, F1 progeny with no direct PFOS exposure, born to parents embryonically exposed to PFOS, also experienced behavioral deficits, which were attributed to maternal transfer of PFOS to eggs (Wang M. et al., 2011; Chen et al., 2013). While concentrations of PFAS measured in adult tissues were often low following developmental exposure, those exposures still caused neurobehavioral toxicity persisting into adulthood (Johansson et al., 2008; Chen et al., 2013). Although only minimally detected in tissues, developmental exposure to PFAS induced a hyperactive phenotype in zebrafish, similar to the changes in motor activity observed in rodents.

Exposure of zebrafish larvae to ≥0.343 µM of 8:8 perfluoroalkyl phosphinic acid (8:8 PFPiA) similarly resulted in decreased overall distance traveled. However, in contrast to other compounds, 5.79 µM 8:8 PFPiA caused a concurrent decrease in locomotor speed during a light-to-dark transition (Kim et al., 2020). A decrease in locomotor speed was also seen in zebrafish larvae exposed to 0.24, 1.2, or 6 mg/L PFDoA, in addition to decreased expression of acetylcholine (Ach) at 6 mg/L and AchE at 1.2 and 6 mg/L (Guo et al., 2018). Gaballah et al. (2020) found that concentrations as low as 0.6 µM PFOS and 3.1 µM perfluoroheptanesulfonic acid (PFHpS) caused hyperactivity in zebrafish, but concentrations up to 80 µM of PFOA, perfluoro-3,6-dioxa-4-methyl-7-octenesulfonic acid (PFESA-1) or 4,8-dioxa-3H-perfluorononanoate (ADONA) caused no observed neurotoxicological impact. These disparate results suggest that structural differences across PFAS compounds may lead to different neurobehavioral consequences of exposure and warrants further investigation. Zebrafish serve as an excellent animal model for higher throughput assessment of the toxic impacts of PFAS, compared to rodent models, and should continue to be used to probe the potential neurotoxicity of diverse classes of PFAS, as defined by chain length and functional group. This approach could facilitate identification and prioritization of specific PFAS classes that can then be further characterized in mice and rats.

Beyond changes in motor activity, impaired learning and memory has also been reported in multiple studies. In Wistar rats, Wang et al. (2015a) found that when dams were exposed to 5 or 15 mg/kg/day PFOS through drinking water during gestation (GD 0—PND 1), lactation (PND 1—35), or perinatally (GD 0—PND 35), male and female offspring experienced impaired performance in a Morris Water Maze task. Offspring in all treatment groups experienced increased latency to escape and increased distance traveled to escape the maze, indicative of impaired spatial memory, with more severe effects in treatment groups exposed during the gestational window (Wang et al., 2015a). In contrast, no significant changes in learning and memory in a T maze delayed alternation task were reported in male and female SD rats at PND 22 after *in utero* exposure to PFOS (Lau et al., 2003). In this study, dams were exposed to 3 mg/kg/day PFOS from GD 2 to GD 21. It is important to note that in this study, only two male and female pups in each treatment group were assessed in the delayed alternation task. Therefore, caution is warranted due to insufficient statistical power, which may contribute to inconsistent findings across studies. Another study conducted in chicks indicated that *in ovo* exposure to 5–10 mg/kg PFOS or PFOA caused decreased scores associated with imprinting behavior, indicative of diminished learning and memory (Horn, 2004; Pinkas et al., 2010).

Some other neurobehavioral endpoints associated with developmental exposure to 6 mg/kg/day PFOS, from GD 12–18, include decreased coordination and motor function, as evidenced by decreased climbing ability, decreased forelimb grip strength, and decreased resistance in a tail pull test in male and female mice (Fuentes et al., 2007a). Similar findings have also been observed in rats exposed to 1 mg/kg/day PFOS from GD 0—PND 20, exhibiting decreased hindlimb grip strength in males, and decreased motor coordination in a rotarod test in both sexes exposed to 10 μg/ml PFOA in drinking water from GD 1—PND 21 (Butenhoff et al., 2009b; Cheng et al., 2013). Finally, *in ovo* exposure to a single dose of 1, 2.5, or 5 mg/kg egg weight of PFOS resulted in brain asymmetry in chickens at post-hatch day 14, although the severity of asymmetry was not dose-dependent (Peden-Adams et al., 2009). Brain asymmetry has been linked to several developmental neurological disorders and deserves further attention in the assessment of PFAS-induced neurodevelopmental abnormalities (Berretz et al., 2020).

Several epidemiological studies have also reported positive associations between PFAS exposure and the prevalence of ADHD or impulsivity in children, however the current evidence base is inconsistent, and is insufficient for inferring causality between developmental exposure to long-chain PFAS and adverse neurobehavioral outcomes humans (Rappazzo et al., 2017; Cao and Ng, 2021). While behavioral findings vary across studies, evidence suggests that exposure to long-chain PFAS leads to impaired motor activity in rodent and zebrafish models, with the most consistent finding across studies being a hyperactive phenotype after exposure to PFAS. Furthermore, these impairments in motor activity seem to persist into adulthood following developmental exposure. While less abundant in the literature, studies have demonstrated that PFOS and PFOA have the capacity to developmentally disrupt learning and memory, as well as motor coordination. However, much of this research has been conducted in zebrafish, and expanding on the current body of literature to include a greater representation of animal models, and chemical classes of PFAS beyond the perfluoroalkyl acids, is necessary to fully understand the neurotoxic potential of developmental exposure to long-chain PFAS.

### Short-Chain PFAS

Data reporting developmental neurotoxicity of short-chain PFAS is sparse, we only identified two experimental *in vivo* studies, and currently available information lacks consistent findings. Similar to PFOS and PFOA, a one-time oral dose of 0.92, 6.1, or 9.2 mg/kg PFHxS at PND 10 caused altered spontaneous behavior in male and female mice at 2 months of age (Viberg et al., 2013). For mice in the high-dose group, this phenotype was accompanied by decreased habituation, and effects persisted at 4 months of age, indicating a long-lasting alteration in motor activity in the high dose group (Viberg et al., 2013). Viberg et al. (2013) also reported that an injection of nicotine at 4 months of age, meant to probe cholinergic system function, caused hyperactivity in control mice, as well as low- and medium-dose group mice. Acting as an agonist at nicotinic cholinergic receptors, nicotine is known to cause an increase in activity in adult NMRI mice. However, this hyperactive phenotype in response to nicotine was absent in mice developmentally exposed to 9.2 mg/kg PFHxS, indicating possible disruption of the cholinergic system (Viberg et al., 2013). PFHxS exposure at concentrations as low as 4.4 µM also induced hyperactivity in zebrafish in a light-dark assay, in addition to 14 µM PFHxA and 3.1 µM perfluropentanesulfonic acid (PFPeS), however no effect to locomotor activity was observed with 100 µM PFBS exposure (Gaballah et al., 2020). Research on developmental exposure to short-chain compounds is clearly lacking, and overlapping evidence between long- and short-chain compounds on neurobehavioral endpoints demonstrates that this area of study needs more focus. Available studies suggest that short-chain PFAS exposure during development can disrupt locomotor activity. However, there is not enough evidence for a clear description of those impacts.

## Neurotoxic Effects of Co-Exposures and Mixtures of PFAS

Mixtures of environmental chemicals often result in toxicity that differs from that of single chemical exposures, due to additive, synergistic, or antagonistic toxicity. A small number of studies have demonstrated that mixtures of PFAS have complex interactions that can cause different toxicological effects than each individual chemical (Ding et al., 2013; Hoover et al., 2019; Preston et al., 2020). Furthermore, co-exposures to PFAS and other toxicants can also result in toxicity that differs from that of either toxicant alone.

### Impacts on Accumulation and Distribution

Exposure to PFAS in combination with other xenobiotics, or particular environmental conditions, has been shown to have differential impacts on xenobiotic metabolism and distribution to tissues throughout the body (Wang F. et al., 2011; Li Y. et al., 2017; Vidal et al., 2019; Bangma et al., 2022). A study conducted by Wang F. et al. (2011) demonstrated that co-exposure to PFOS and Pentabromodiphenyl ether (BDE-47), a brominated flame retardant known to cause developmental neurotoxicity, caused decreased serum and brain concentrations of both chemicals compared to exposure to each chemical individually, in Wistar rat dams and pups when dams were exposed from GD 1 to PND 14. One possible explanation offered by the authors for these decreased toxicant concentrations in the serum and brain is that both chemicals dose-dependently activate xenobiotic metabolizing enzymes, and the interaction between the two may alter this enzyme activation (Wang F. et al., 2011). While potentially important for BDE-47, an increase in xenobiotic metabolism is not likely to be relevant for PFOS concentrations in the body, as PFAAs are resistant to enzymatic metabolism (Kudo and Kawashima, 2003; Lau et al., 2007). Other important considerations that were not discussed or directly explored in this study are changes in the expression of transport proteins and competition for transport across the BBB, which could reduce transport capacity as a whole and limit uptake of both BDE-47 and PFOS. Wang F. et al. (2011) also found that co-exposure to PFOS and BDE-47 cause timepoint-dependent and brain-region-dependent effects on mRNA expression of brain-derived neurotrophic factor (BDNF), a gene that encodes a protein involved in regulation of neuronal growth and development.

Another study demonstrated that co-exposure to PFOS and single-walled carbon nanotubes (SWCNT), graphene-based biomaterials, altered the bioaccumulation of PFOS in adult zebrafish (Li Y. et al., 2017). Zebrafish exposed to PFOS and SWCNT accumulated less PFOS in the brain, liver, intestines, and gills, compared to fish exposed only to PFOS, and concentrations of PFOS in these tissues decreased with increased doses of SWCNT at every time point measured (Li Y. et al., 2017). In addition, fish in the co-exposure group exhibited increased PFOS accumulation in the skin, which authors attributed to PFOS adsorption by SWCNT, decreasing bioavailability to internal organs and increasing adherence to epithelial surfaces (Li Y. et al., 2017). This finding agreed with an *in vitro* study, which demonstrated that SWCNT had high sorption capacity for PFOS (Chen et al., 2011). Despite the observed PFOS sequestration in the skin of fish co-exposed to PFOS and SWCNT, the authors found the greatest integrated biomarker response (IBR), an index utilizing multiple biomarker measurements to predict organismal stress caused by environmental contaminants, in the brain and other internal organs of co-exposed fish after 24 h of exposure (Broeg and Lehtonen, 2006; Li Y. et al., 2017). In this study, Li Y. et al. (2017) utilized measures of oxidative stress and AchE activity in their IBR model. However, this enhanced IBR seen after 24 h in the co-exposure treatments did not persist at other time points as the study continued (Li Y. et al., 2017). These studies demonstrate that co-exposures can alter bioavailability and tissue distribution of PFAS, even decreasing exposure in certain tissues, which may not necessarily correspond to reduced toxicity.

Distribution of PFAS throughout the body can also be impacted by abiotic factors, including salinity and temperature, which may alter the levels of proteins that bind PFAS (Bangma et al., 2022). For example, Vidal et al. (2019) found that in a 28-day dietary exposure experiment in adult rainbow trout, concentrations of PFOS and PFHxS in the blood, liver, and brain increased with increased water temperature, as did the brain to blood ratio for PFOS. Vidal et al. (2019) reported different elimination half-lives for PFOS and PFHxS in the brains of fish held at different incubation temperatures, determining that half-lives for both compounds were significantly shorter in 7 and 19°C waters, than in the optimal temperature range for rainbow trout, at 11°C. Authors predicted that in addition to altered elimination rates, the temperature-mediated increase in PFAS accumulation in the liver and brain could be due to increased cardiac output in warmer temperatures resulting in altered perfusion of these organs, as observed in another study assessing rainbow trout blood flow (Barron et al., 1987; Vidal et al., 2019). These data, which are primarily relevant to PFAS distribution in wildlife, highlight the importance of investigating PFAS exposure and neurotoxicity in varying environmental conditions, as these factors could influence PFAS accumulation in the brain, particularly in the wake of climate change (Borgå et al., 2010; Houde et al., 2011; Vidal et al., 2019).

### Neurological Consequences of Mixtures

To our knowledge, only six *in vivo* studies have assessed the neurological consequences of exposure to mixtures of PFAS, or mixtures including PFAS and other toxicants. A mesocosm study in Northern leopard frogs (*Rana pipiens*) compared neurotransmitter levels in frog brains after larval exposure to 10 ppb PFOS or 10 ppb of a PFAS mixture, assessing one subset after 30 days of exposure and another after frogs reached metamorphosis (Foguth R. M. et al., 2020). The PFAS mixture contained PFOS, PFHxS, PFOA, PFHxA, and PFHpA, in a ratio mimicking that measured in surface water at Clark’s Marsh, an aqueous film forming foam (AFFF) exposed site in Michigan (Foguth R. M. et al., 2020). Foguth et al. (2020b) found that after a 30-days exposure, glutamate levels in the brain were significantly reduced in both exposure groups compared to control animals, however serotonin levels were reduced only in the brains of frogs exposed to the mixture. At metamorphosis, these changes in glutamate and serotonin in the brains of exposed frogs did not persist, however acetylcholine levels were significantly increased in both exposure groups compared to controls (Foguth R. M. et al., 2020). In another study, A/J mice were exposed *via* diet from 3–13 weeks of age to environmentally relevant concentrations of a mixture of eight PFAS (PFOS, PFOA, PFNA, PFUnDA, PFDoDA, PFTriDA, and PFTeDA), based on concentrations measured in earthworms in Trondheim, Norway (Grønnestad et al., 2021). Exposure to this mixture of PFAS caused decreased brain dopamine levels in male mice only, along with decreased levels of tyrosine hydroxylase, an enzyme necessary for dopamine synthesis, and in female mice this mixture caused an increase in brain expression of Dr2 dopamine receptors (Grønnestad et al., 2021). Grønnestad et al. (2021) did not assess the impacts of each compound individually, so more information is needed to determine whether each component of this chemical mixture behaves differently than the sum of its components.

Two studies have investigated the neurodevelopmental impacts of co-exposure to PFAS and methylmercury (MeHg), a well-characterized neurotoxicant, reporting similar behavioral findings for several endpoints (Cheng et al., 2013; Reardon et al., 2019). Cheng et al. (2013) investigated the combined effects of 10 ppm PFOA and 10 ppm MeHg drinking water exposure in Wistar rat pups from GD 1 to PND 21 and compared to 10 ppm PFOA or MeHg alone. In another study, Reardon et al. (2019) exposed SD rats *via* diet from GD 1 to PND 1, to either 1 mg/kg/day PFOS, 1 mg/kg/day MeHg, or a low dose (0.1 mg/kg PFOS +1 mg/kg MeHg) or high dose (1 mg/kg PFOS +1 mg/kg MeHg) mixture of the two toxicants. In both studies, hyperactivity in an open field was observed in single chemical exposures, but absent in co-exposure treatment groups (Cheng et al., 2013; Reardon et al., 2019). Cheng et al. (2013) found that exposure to PFOA, MeHg, or both toxicants impaired motor coordination in a rotarod test in comparison to control animals. However, Reardon et al. (2019) found that while decreased motor coordination was observed in exposure to PFOS or MeHg alone, this effect too was absent in co-exposed rats. These behavioral findings were reinforced in a metabolomic analysis of the rat prefrontal cortexes, with hierarchical clustering data indicating that MeHg-only and PFOS-only exposed rats had altered metabolomic profiles, in contrast with control and mixture exposed profiles which clustered together (Reardon et al., 2019). In contrast, Reardon et al. (2019) did observe that newborn pups in the high-dose mixture treatment group exhibited some unique delays in neurobehavioral development, and only mixture exposed juvenile rats exhibited decreased anxiety in an elevated plus maze (Reardon et al., 2019). Collectively, these studies illustrate the PFAS exposure in conjunction with a known neurotoxicant, MeHg, can impact brain function in a way that is unique from exposure to PFAS or MeHg alone. Authors hypothesized that PFOA and PFOS may induce conformational changes in muscarinic cholinergic receptors, such as those induced by polychlorinated biphenyls (PCBs), thereby altering MeHg binding sites and impairing retention of mercury in the brain (Coccini et al., 2007; Cheng et al., 2013; Reardon et al., 2019). While co-exposure to PFOA/PFOS and MeHg seems to attenuate some of the behavioral impairments induced by each toxicant alone, this phenotype may be transient and the long-term implications of this co-exposure are not yet known.

Finally, two studies have reported on neurological outcomes in zebrafish and mice following exposure to a more diverse mixture of environmental contaminants, including PFAS. A study conducted by Khezri et al. (2017) assessed the effects of seven different mixtures of POPs on zebrafish behavior following embryonic exposure from 6–48 h post fertilization (hpf) or 48–96 hpf. Mixtures of POPs in this study were selected based upon measured levels of contaminants in human plasma from Scandinavian study participants, and consisted of mixtures of PFAS, polybrominated diphenyl ethers (PBDEs), polychlorinated biphenyls (PCBs), and other organochloride contaminants (Khezri et al., 2017). At concentrations 20 × those measured in the Scandinavian cohort, Khezri et al. found that zebrafish exposed to the mixture containing all the POPs from 48–96 hpf experienced an increase in swimming speed at 96 hpf compared to controls (2017). However, all mixtures containing PFOS elicited the same effect at this time point and did not exceed the effect observed in fish exposed to PFOS alone, indicating that PFOS was the driving factor in this behavioral response (Khezri et al., 2017).

The second study assessed the impact of a mixture of four different environmental toxicants, PFOA, atrazine (ATR), bisphenol-A (BPA), and tetrachlorodibenzodioxin (TCDD) that have been found to disrupt endocrine function and have been associated with neurobehavioral changes (Sobolewski et al., 2014). In this study, C57BL/6 mice were exposed to a mixture of these four chemicals, or each individually, from GD 7 to PND 1, and the neurodevelopment of their offspring was assessed in a behavioral battery (Sobolewski et al., 2014). In this study, sex-specific mixture effects were observed, as only male mice that were exposed to the chemical mixture exhibited significantly diminished short-term memory in a novel object recognition task, and increased response rates in a fixed interval (FI) schedule of reward task, indicative of increased impulsivity (Sobolewski et al., 2014). Interestingly, animals exposed individually to three of the chemicals in the mixture (ATR, BPA, or TCDD) experienced decreased response rates in the FI task, highlighting a potentially unique additive effect in the mixture exposed animals (Sobolewski et al., 2014). However, some results also suggested that chemicals in the mixture can have counteracting effects for a few endpoints, as a hyperactive phenotype was only observed in PFOA-exposed male mice, while an increased FI response rate occurred only in TCDD-exposed female mice (Sobolewski et al., 2014).

Analysis of chemical mixtures is an important area of research, as humans and wildlife are most likely to be exposed to toxicants as low-dose mixtures. Therefore, analysis of the neurotoxicity of mixtures containing PFAS are potentially the most relevant and important studies for understanding the impacts of these toxicants on the brain in the general population. However, there are exceedingly few studies that have looked at the neurotoxicological effects of mixtures containing PFAS. This is a critically important area of research that needs more attention.

## Neurophysiological and Neurochemical Mechanisms Driving PFAS Toxicity

Despite the inconsistencies in experimental design and results across studies, it is clear that PFAS can accumulate in and subsequently impact gross brain function. Limited information is available regarding the impact of PFAS structure on neurophysiological and neurochemical mechanisms. However, we did find two studies that reported structure related differences in toxicity which indicate that disruption of neuronal activities seems to increase with carbon chain length and fluorination level, and highlight perfluorinated sulfonates as more neurotoxic than carboxylates (Liao et al., 2009; Liu et al., 2011). Continued investigation into differences in toxicity because of carbon chain length, level of fluorination, and functional group are needed.

At the molecular level, several mechanisms of PFAS-, primarily PFOA- and PFOS-, induced neurotoxicity have been proposed. Three general mechanisms have received significant attention and have recently been reviewed by Cao and Ng (2021) and Piekarski et al. (2020). These include changes in calcium homeostasis, disruption of neurotransmitters, and neuroendocrine dysregulation. Here, we briefly summarize the significance of these molecular changes for neuron, circuit, and overall brain function and highlight other underappreciated and indirect mechanisms of PFAS neurotoxicity that warrant further investigation.

### Alterations in Calcium Handling and Homeostasis

**FIGURE 5:**
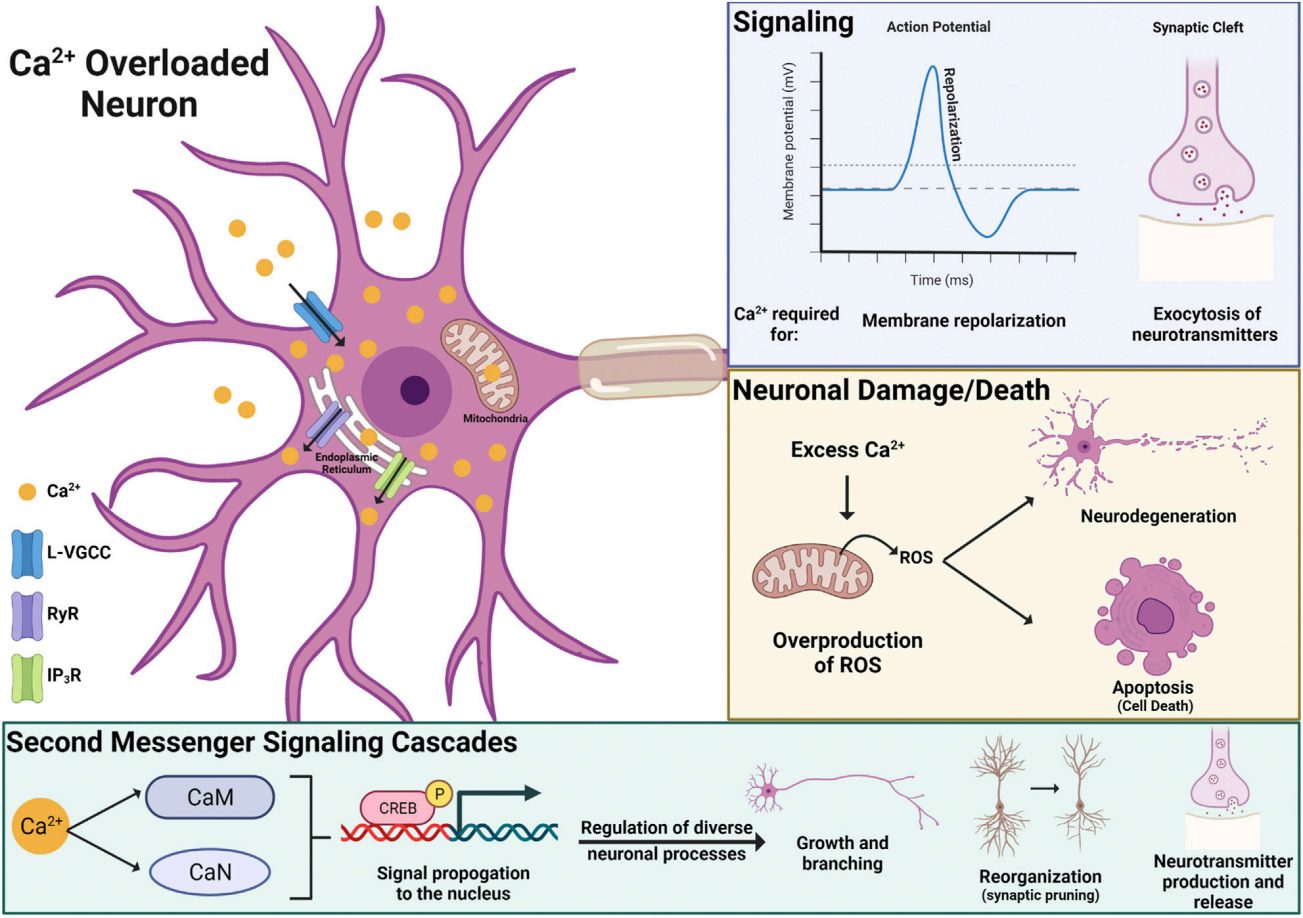
Mechanisms driving PFAS-induced neurotoxicity include direct mechanisms, such as disruption of calcium (Ca^2+^) homeostasis. PFAS-associated Ca^2+^ overload in neurons appears to be driven by Ca^2+^ influx from the extracellular space, through L-type voltage-gated Ca^2+^ channel (L-VGCC), and intracellular Ca^2+^storage organelles (mitochondria and endoplasmic reticulum), *via* inositol 1,4,5-triphosphate receptors (IP3R) and ryanodine receptors (RyR). Superfluous Ca^2+^ can disrupt neuronal signaling, induce oxidative stress leading to neuronal cell death, and disrupt Ca^2+^ dependent second messenger signaling cascades (CaM: calmodulin, CaN: calcineurin) that regulate diverse neuronal cell processes, including growth, reorganization, and the production and release of neurotransmitters.

The activity of excitable cells, like neurons, can be drastically impacted by disruptions in intracellular ion homeostasis. There are some data showing that PFOS can impact gating properties of sodium and potassium channels, ions critical for forming electrical impulses (i.e., action potentials). However, these studies only report significant effects at high concentrations, above 30 µM, and cannot rule out nonspecific membrane impacts (Harada et al., 2006). The most consistently reported ion to be disrupted by PFAS exposure is calcium (Ca^2+^). PFAS-induced increases in neuronal Ca^2+^ have been observed in both *in vivo* and *in vitro* studies and appear to be driven by Ca^2+^ influx from the extracellular space and intracellular Ca^2+^ storage organelles, such as the mitochondria and endoplasmic reticulum (Dusza et al., 2018; Fang et al., 2018; Liao et al., 2008; Liu et al., 2011; Wang et al., 2015b). More specifically, *in vitro* pharmacological studies have identified the L-type voltage-gated Ca^2+^ channel, inositol 1,4,5-triphosphate receptor, and ryanodine receptors as mediators of Ca^2+^ overload in PFAS exposed neurons (Figure 5; Liao et al., 2008; Liu et al., 2011). However, the role of other Ca^2+^ channels and receptors and how PFAS are interfacing with these proteins remains to be elucidated.

Beyond its involvement in transmitting information about depolarization status, Ca^2+^ is a critical second messenger that initiates numerous signaling cascades, mediating diverse neuronal processes including regulation of gene expression, plasticity (i.e. growth and reorganization of neurons), and neurotransmitter secretion (Figure 5). Several Ca^2+^ dependent signaling molecules, including Ca^2+^/calmodulin-dependent protein kinase II (CaMKII), cAMP-response element binding protein (CREB), and calcineurin (CaN) have also been shown to be sensitive to PFAS exposure (Dusza et al., 2018; Harada et al., 2006; Liu et al., 2011, 2010a, 2010b). *In vitro* studies using cultured rat neurons have shown PFOS- and PFOA-induced increases in Ca^2+^ levels and expression of CaN, a downstream signaling molecule that plays an important role in synaptic plasticity, cell survival, and cognition. These findings were more pronounced with PFOS exposure, as the magnitude of increase was significantly greater and observed at lower exposure concentrations for PFOS (30 µM) than PFOA (100µM; Liu et al., 2011). Additionally, an *in vivo* study using Sprague-Dawley rats reported increased expression of CaMKII and CREB in the cortex and hippocampus of animals exposed to 15 mg/L (∼30 µM) PFOS, highlighting Ca^2+^ dependent signaling cascades as a mechanism that is vulnerable to PFOS exposure *in vitro* and *in vivo*.

Disruptions in Ca^2+^ homeostasis and its signaling pathways likely contribute to other PFAS associated changes in neurophysiology, including alterations in neuron structure, signaling, and even neuronal cell death (Wan Ibrahim et al., 2013; Foguth R. M. et al., 2020; Di Nisio et al., 2022). For example, increases in reactive oxygen species (ROS) in Ca^2+^ overloaded neurons and mitochondrial dysfunction in PFOS and PFOA exposed brains have also been observed (Liu et al., 2011; Salimi et al., 2019). Ca^2+^ plays an essential role in activating the citric acid cycle and stimulating ATP production through the respiratory chain within mitochondria. Under physiological conditions the ROS produced by these processes are not harmful to the cell. However, excess Ca^2+^ can result in overproduction of ROSs, inducing oxidative stress within neurons and ultimately leading to cell death. Because of its diverse role within cells, superfluous Ca^2+^ in PFAS exposed neurons could greatly alter neuronal function and even lead to neurodegeneration, a neuropathology frequently observed in progressive diseases like Alzheimer’s, for which PFAS exposure has been associated with increased risk (Mastrantonio et al., 2018). Further interrogation of Ca^2+^ induced neurotoxicity should include PFAS from diverse structural classes to improve our understanding of which congeners may pose the greatest risk to long-term adverse health outcomes, such as neurodegenerative diseases like Alzheimer’s.

### Neurotransmitters

Neurotransmitters are signaling molecules created and stored within neurons that are released at the synapse in response to an action potential and are ultimately responsible for perpetuating signals in the brain. Levels of these chemical messengers within the brain are thus related to neuronal activity and signal transmission. PFAS exposure has been shown to disrupt glutamate, γ-aminobutyric acid (GABA), dopamine, acetylcholine, and serotonin levels (Long et al., 2013; Hallgren et al., 2015; Li Z. et al., 2017; Sammi et al., 2019; Foguth R. M. et al., 2020). Several of these neurotransmitters, specifically dopamine, acetylcholine, and serotonin are chemical messengers for essential diffuse modulatory circuits, which are made up of a core set of neurons that can regulate the activity of many other neurons in the brain. In other words, changes in signaling associated with these neuromodulators are likely to have broad, wide-reaching implications for brain function and behavior, such as the PFAS-induced impairments in learning, memory, and feeding behaviors reviewed in *Neurotoxic Effects of Adult Exposure to PFAS, Neurotoxic Effects of Developmental Exposure to PFAS*, and *Neuroendocrine Dysregulation*.

The dopaminergic system has received the most attention due to its apparent sensitivity to PFAS, with dopaminergic neuropathology observed at lower exposure levels compared to other neurotransmitter systems and consistent disruption across model organisms (*M. musculus*, *D. rerio*, *R. pipiens*, *C. elegans*, etc.) (Foguth et al., 2019; Foguth R. M. et al., 2020; Grønnestad et al., 2021). For example, a study in *C. elegans* demonstrated that exposure to PFOS causes dopaminergic neurotoxicity at 50 µM, whereas GABAergic, cholinergic, and serotonergic neurons did not show neurotoxicity at concentrations below 200 µM PFOS. The authors also noted that mitochondrial content was significantly reduced, and ROS levels were increased by PFOS at lower exposure concentrations, 2 and 5 µM, respectively, than those required to induce dopaminergic neuropathology. Therefore, mitochondrial dysfunction and increased ROS production may be an underlying mechanistic driver of dopaminergic neurotoxicity and loss of dopaminergic neurons (Sammi et al., 2019).

There are inconsistencies in the literature regarding the direction of change for dopamine levels with dopamine increasing with PFAS exposure in some studies and decreasing in others (Long et al., 2013; Salgado et al., 2016; Yuan et al., 2018). Some of these differences are likely the result of dissimilarities in dose, age of exposure, time since exposure, and species across studies. However, it is also important to recognize that brain structure and function is region-specific and these inherent complexities, along with region-specific accumulation of PFAS as discussed earlier, can contribute to multifaceted findings. For example, within the same study expression of dopamine receptors changed with PFOS exposure in a subtype-specific (D_1_-like versus D_2_-like receptors) and brain region-specific manner, across the amygdala, hippocampus, and cortex (Salgado et al., 2016). Disruption of neurotransmitters and neuromodulators by PFAS may contribute to increased risk of neuropsychiatric disorders, as well as other health risks associated with PFAS exposure, such as obesity, cancer, and thyroid disease, as these chemical messengers regulate diverse physiological processes and organ systems (Blake and Fenton, 2020; Fenton et al., 2021).

### Neuroendocrine Dysregulation

Both human and animal studies demonstrate that PFAS can act as endocrine disruptors with wide-ranging effects on neurological function, behavior, and vulnerability to disease (Piekarski et al., 2020). The brain is essential for regulating the synthesis and release of hormones throughout the body, with the hypothalamus acting as the primary coordination center for many of these hormones. The majority of neuroendocrine research focuses on regulation of stress, sex, and thyroid hormones, largely due to their critical roles in health and disease risk including neuropsychiatric, metabolic, cardiovascular, and reproductive diseases. Stress, sex, and thyroid hormones are all regulated by the brain through a similar three stage hierarchy involving the hypothalamus, pituitary, and respective target endocrine organ, in this case adrenals, gonads, or thyroid. A recent review by Piekarski et al. (2020) provides further detail on these axes and PFAS-associated dysregulation, highlighting thyroid hormone as the most extensively studied. PFAS could act at any or all of these three levels, and further studies are needed to untangle these complexities, as feedback and feedforward loops, as well as compensatory mechanisms, are involved in neuroendocrine signaling.

A majority of evidence for PFAS-induced endocrine disruption is at the level of circulating stress, sex, and thyroid hormones, while data regarding PFAS associated disruption of synthesis enzymes and receptors is more limited, especially within the brain (Pereiro et al., 2014; Wang et al., 2014; Webster et al., 2014; Berg et al., 2015; Itoh et al., 2016; Yang et al., 2016; Goudarzi et al., 2017; Wang H. et al., 2019). One finding that is worth noting is the reduced expression of kisspeptin and its receptor in the hypothalamus of mice and rats exposed to 10 mg/kg PFOS and PFOA (Wang X. et al., 2018; Du et al., 2019). Kisspeptins are proteins produced in the brain, most notably in the hypothalamus, and are important for initiating the secretion of gonadotropin-releasing hormone from the hypothalamus to act on the pituitary, a regulatory step within the hypothalamic-pituitary-gonadal axis. Wang X. et al. (2018) also reported impaired reproductive activity, with exposed females showing a prolonged estrous cycle and reduced ovulation, findings that are commonly associated with reproductive senescence. While these studies focused on kisspeptin in the hypothalamus, kisspeptins are also expressed in the hippocampus and amygdala. More work is needed to further elucidate the impact of PFAS on neuroendocrine disruption in brain regions outside the hypothalamus, and disruption of other hormone systems, including leptin, insulin, oxytocin, and vasopressin.

Particular attention should be paid to changes in hormonal regulation of metabolism as several studies have observed alterations in food consumption, weight gain, and relevant molecular changes at the level of the hypothalamus. It has been reported in many studies that exposure to PFAS, primarily PFOS and PFOA, can lead to decreased food consumption and decreased weight gain in adult animals (Seacat, 2002; Austin et al., 2003; Thibodeaux et al., 2003; Fang et al., 2008; Butenhoff et al., 2009a, 2012). Although there could be several reasons for decreases in food intake and weight loss, feeding behavior is mainly regulated by hormonal communication with the hypothalamus (Kishi and Elmquist, 2005). Coupled with the report of decreased weight gain and food consumption, Austin et al. (2003) found that PFOS accumulation in the hypothalamus was associated with increased concentrations of serum leptin, a hormone primarily produced by adipose tissue that travels to the hypothalamus to inhibit food intake, thus linking this altered feeding behavior to HPA axis alterations. It is important to note that the impacts of PFAS on body weight are not consistent in the literature, with many studies reporting associations between PFAS exposure and increased body weight gain and metabolic syndrome-like phenotypes in human and rodent studies (Hines et al., 2009; Halldorsson et al., 2012; Braun et al., 2021; Cope et al., 2021).

The first study to directly investigate the role of the hypothalamus in PFOS and PFOA-induced disruptions in feeding behavior was conducted by Asakawa et al. (2007). It was shown that when administered through intracerebroventricular (ICV) injection in mice, PFOS and PFOA caused a dose-dependent decrease (Asakawa et al., 2007; Camilleri, 2015) in food intake, with effects lasting 24 h. The group also found that PFOS decreased gastric emptying in mice and influenced gastroduodenal motility in rats, two functions that are controlled through feedback mechanisms on highly organized hypothalamic circuitry, particularly the arcuate nucleus of the hypothalamus (Asakawa et al., 2007; Browning and Travagli, 2014; Camilleri, 2015). In the hypothalamus, PFOS caused upregulation of urocortin 2, an endogenous ligand for corticotropin releasing factor receptor 2 (CRFR2) that has been shown to suppress feeding behavior and inhibit gastric emptying (Reyes et al., 2001; Czimmer et al., 2006). Feeding behavior was rescued through administration of a CRFR2 antagonist, thus validating the role of CRFR2 activity in the impaired behavior (Asakawa et al., 2007). While ICV administration of PFOS and PFOA yields very high concentrations in the brain, thus making the exposure unrealistically high compared to human exposures, these results provide mechanistic insight regarding ways in which high-dose PFAS exposure may influence feeding *via* the hypothalamus. A follow up study by Asakawa et al. (2008) examining the activity of PFOA on mice, administered *via* intraperitoneal (IP) injection, yielded similar findings with decreases in food intake and delayed gastric emptying. In addition, they saw an increase in hypothalamic paraventricular nucleus (PVN) concentrations of an endogenous ligand that has been shown to attenuate feeding and gastric motility, urocortin 1, in mice exposed to PFOA (Asakawa et al., 2008). Taken together, this information indicates that high concentrations of PFOS and PFOA can impact the hypothalamus and endocrine regulation of metabolic pathways.

### Indirect Toxicity and PPARs

**FIGURE 6:**
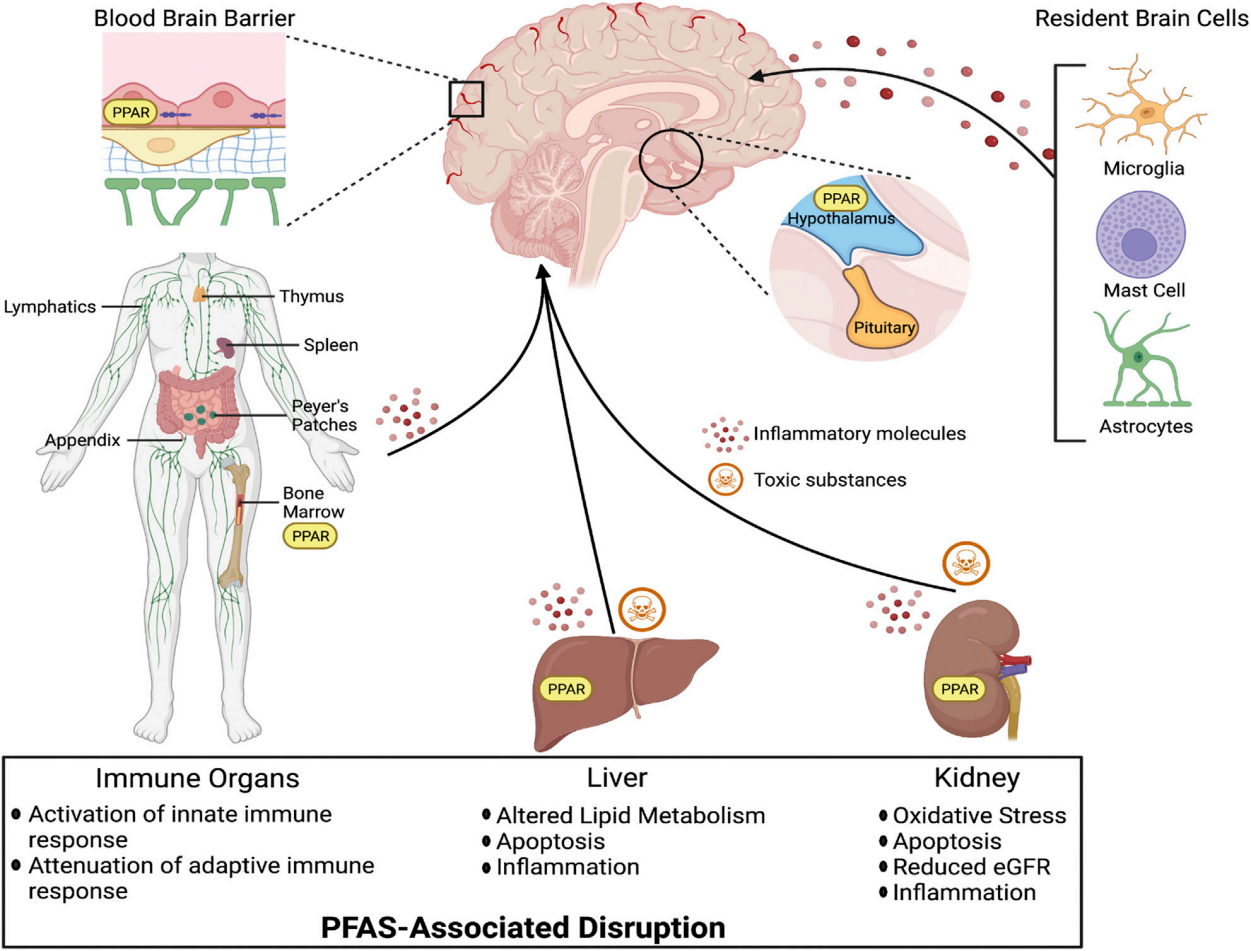
Important indirect mechanisms by which PFAS exposure may impact neurological health include disruption of liver, kidney, and peripheral immune system functions. Build up of toxic substances and inflammatory molecules in circulation have the potential to compromise the BBB, damage neurons, and contribute to neurodegenerative diseases. PFAS have also been identified as PPAR agonists which are expressed in the liver, kidneys, immune organs, and brain, making PPARs an important molecular target for both direct and indirect effects of PFAS in neurological health.

Beyond the three mechanisms described so far, there are important indirect impacts of PFAS that should be taken into consideration in the context of neurotoxicity (Figure 6). The brain is connected to and influenced by many other organs and organ systems in the body, including the liver, kidney, and immune system. Neuropsychiatric conditions, such as depression, anxiety, psychosis, and cognitive impairment, are often prevalent in patients with chronic kidney disease (CKD), non-alcoholic fatty liver disease (NAFLD), and autoimmune disorders (Jeppesen and Benros, 2019; Simões e Silva et al., 2019; Soto-Angona et al., 2020). Therefore, toxicity and disrupted function of these organ systems by PFAS may have significant repercussions for brain function and long-term neurological health.

The liver and kidney are essential for detoxification and elimination of toxins from the body, including potentially toxic byproducts like ammonia produced during protein digestion. PFAS exposure has been associated with dysfunction and genesis of kidney and liver disease, including increased risk for carcinogenesis in these tissues (Stanifer et al., 2018; Bassler et al., 2019). Other PFAS-associated kidney and liver diseases identified in the literature include increased risk of CKD and NAFLD. Toxicology studies, including *in vivo* and *in vitro* experiments, have identified histological and cellular outcomes indicative of PFAS-induced nephrotoxicity (Shankar et al., 2011; Stanifer et al., 2018). Such findings include oxidative stress, apoptosis, and enhanced microvascular endothelial permeability. Human studies have also established a link between PFAS exposure and disruptions in kidney function, with estimated glomerular filtration rate (eGFR), a blood test that estimates the volume of blood filtered by the kidneys (≥90 indicates healthy kidneys), as the primary method for monitoring kidney disease. Multiple studies have reported significant associations between PFAS exposure with lower eGFR and increased odds of CKD (Shankar et al., 2011; Vearrier et al., 2013; Watkins et al., 2013; Kataria et al., 2015; Dhingra et al., 2017; Blake et al., 2018; Stanifer et al., 2018).

Similar to PFAS-associated kidney dysfunction, animal and human studies have highlighted the liver’s sensitivity to PFAS. For example, animal studies have identified disruptions in hepatic lipid metabolism and induction of apoptosis in the liver with PFAS exposure, molecular changes commonly observed in patients with NAFLD (Kim et al., 2011; Rebholz et al., 2016). Human cohorts have shown positive associations between serological biomarkers of hepatocyte death/apoptosis and serum PFAS concentrations (Bassler et al., 2019). Collectively, these studies have called attention to the liver and kidney as important target tissues of PFAS toxicity. Reduced functional capacity of the liver and kidneys may contribute to the neurotoxic effects of PFAS exposure through the buildup of toxic substances in circulation that can cross and/or impact the function and integrity of the BBB.

The mechanisms of PFAS-induced immunotoxicity are poorly understood, however evidence from experimental animal and human epidemiologic studies have linked increased PFAS exposure with altered immune functions. For example, elevated lysozyme activity, an antimicrobial enzyme and important biomarker of innate immune system function, has been reported in both mammalian and non-mammalian species exposed to PFAS (Guillette et al., 2020; Peden-Adams et al., 2009, 2008). Furthermore, *in vivo* and *in vitro* experiments have demonstrated that PFAS exposure can lead to severe inflammation in organs, including the liver, kidneys, and nervous system (Qian et al., 2010; Dong et al., 2012; Qazi et al., 2013; Xing et al., 2016; Xu et al., 2019; Wang et al., 2021). Systemic inflammation can disrupt the integrity of the BBB through modification of tight junctions, endothelial damage, degradation of extracellular matrix components, and changes in astrocytes (Varatharaj and Galea, 2017). Therefore, immune crosstalk between the liver, kidneys, and brain may be an underappreciated mechanism by which PFAS exposure contributes to neurotoxicity (Figure 6).

In addition to these indirect mechanisms, the brain contains resident immune cells, such as microglia and mast cells, along with astrocytes, oligodendrocytes, and neurons, that produce and respond to inflammatory molecules in response to injury, infection, and exposure to environmental contaminants (Rogers et al., 2013; Mokarizadeh et al., 2015; Rock and Patisaul, 2018; Arambula and McCarthy, 2020). As previously discussed, PFAS have been shown to accumulate in the brain meaning they have the potential to activate the brain’s innate immune system. Persistent neuroinflammation can weaken the BBB, damage neurons, and contribute to neurodegenerative diseases, like Alzheimer’s (Gelders et al., 2018).

PFAS exposure may also leave the brain vulnerable to the deleterious impacts of infections, such as COVID-19 (severe acute respiratory syndrome coronavirus 2, SARS-CoV-2), through attenuation of the adaptive immune system (Grandjean et al., 2020; Catelan et al., 2021). The adaptive immune system is a critical subsystem of the immune network that is composed of specialized cells that mount a targeted response to a particular pathogen in order to eliminate and prevent its growth. A number of studies have demonstrated that elevated concentrations of PFAS can lead to immunosuppression, increased severity of infections, and decreased response to vaccines (Grandjean et al., 2012; Granum et al., 2013; Kielsen et al., 2016; Grandjean et al., 2020). While more work is needed to better describe the impact of PFAS on both the innate and adaptive immune system, in the periphery as well as the CNS, current evidence suggests that alterations in immune signaling are a significant risk factor for PFAS-induced toxicity, including neurotoxicity.

Finally, one important molecular target that we have not yet discussed is a group of nuclear receptors known as peroxisome proliferator-activated receptors (PPARs). These nuclear receptors serve as transcription factors and come in three different types PPAR α, γ, and β/δ, which have differences in tissue distribution and the gene sets they control. More specifics on tissue distribution and gene regulation can be found in recent reviews by Hong et al. (2019) and Kirk et al. (2021). As a generalization, PPARs are concentrated in tissues with high metabolic demand, including the liver, kidneys, and brain, and serve as important regulators of the immune system (Zhang and Young, 2002; Hong et al., 2019; Kirk et al., 2021). Therefore, PPARs are an important molecular target that may be relevant to PFAS-induced kidney, liver, and immune disruption with the potential to impact the brain in both a direct and indirect manner.

PPARs play essential roles in regulating cellular differentiation, proliferation, and metabolic pathways, including carbohydrate, lipid, and protein metabolism. *In vivo* and *in vitro* studies have identified individual PFAS as agonists for all three types of PPARs. For example, PFOA has been shown to interact with PPARα, γ, and β/δ and induce adipogenesis in 3T3-L1 cells, cells with a fibroblast morphology that can differentiate into a cell with an adipocyte-like phenotype (Takacs and Abbott, 2007; Watkins et al., 2013; Li et al., 2019). Furthermore, animal studies have demonstrated PFAS associated disruption of hepatic lipid metabolism, which may be mediated, in part, through interactions with PPARs and contribute to increased risk of NAFLD associated with PFAS exposure (Kim et al., 2011; Rebholz et al., 2016; Bassler et al., 2019; Kirk et al., 2021). Metabolic processes are also tightly controlled by the brain, particularly *via* the neuroendocrine system coordinated by the hypothalamus. PPARα is expressed in the PVN of the hypothalamus, where it plays a role in the feedback system that regulates feeding behavior (Asakawa et al., 2008). Therefore, PPARs within the brain may serve as important molecular targets of PFAS that can impact metabolic homeostasis (Ammazzalorso et al., 2019; Behr et al., 2020).

PPARα is expressed at the BBB where it controls the expression of ATP-driven drug efflux transporters, such as P-glycoprotein (Abcb1), breast cancer resistance protein (Bcrp/Abcg2), and multidrug resistance-associated protein (Mrp2/Abcc2) (More et al., 2017). A study from 2017 using rats and mice found that PPARα agonists, including PFOS and PFNA, increased the expression and activity of these efflux transporters. While this may be a protective mechanism, meant to limit the amount of PFAS that can enter the brain, it is important to keep in mind that this may also limit the transport of drugs needed to treat neurological disorders into the brain. Therefore, PFAS exposure may have important implications for the treatment of certain ailments, such as brain cancer, epilepsy, and depression (More et al., 2017; Kirk et al., 2021), through restriction of drug delivery. More work is needed to understand the role of PPARs in both direct and indirect PFAS-induced impacts on brain function and neurological health.

## Neurotoxicity of PFAS in Sensitive Populations

**FIGURE 7:**
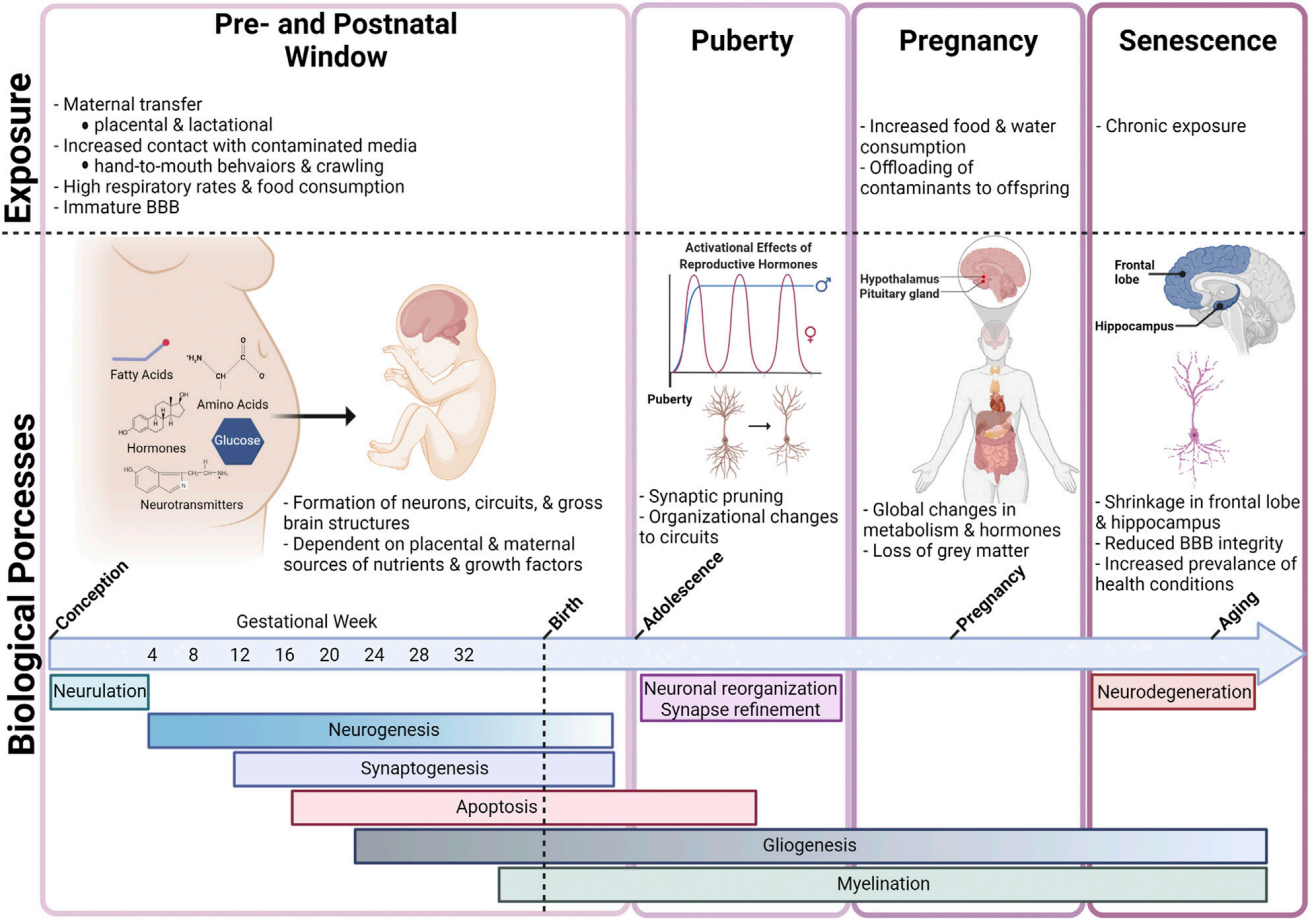
Throughout a person’s lifetime there are several critical windows during which the brain is particularly vulnerable to chemical insult, including the pre- and postnatal period, puberty, pregnancy, and senescence. These windows are characterized by unique exposure profiles and/or the occurrence of dynamic physiological changes that make the brain more plastic or penetrable, and therefore more susceptible to chemical insult.

Populations that are well recognized as being particularly sensitive to PFAS exposure include individuals living near or working in a PFAS manufacturing facility, as well as fetuses, infants, and people who have certain health conditions and/or are immunocompromised. Additionally, critical windows for brain development and maturation, including puberty, pregnancy, and senescence, may result in increased susceptibility to deleterious effects of PFAS. What makes these populations so vulnerable to PFAS are factors that lead to higher-than-average PFAS exposure and/or complex biological processes that have important ramifications for neurophysiology (Figure 7). These conditions can confer heightened risk to individuals in a state-dependent manner, in which the baseline developmental or health status of an individual will be a key modifier of their response to contaminant exposure.

Fetuses and infants are likely to experience high PFAS exposure relative to their body weight during a time when neurons, circuits, and gross brain structures are being established, which may have severe long-term consequences for brain structure and function. In addition to developmental susceptibility, the brain is also more vulnerable to toxic insults during periods of extreme hormonal fluctuation, such as puberty and pregnancy. Hormones play a critical role in neural plasticity and modulate neurophysiological changes that allow us to adapt to our surroundings. For example, synaptic pruning, or loss of gray matter, in regions of the brain involved in social cognition during pregnancy may streamline neural circuits involved in maternal care and behaviors (Hoekzema et al., 2017). Exposure to PFAS, which have neurotoxic and endocrine disrupting properties, during these windows could interfere with these hormonally driven processes. Finally, senescence is an important period of vulnerability for the brain, because as we age, we experience chronic exposure to persistent chemicals (like PFAS), undergo normal age-related deterioration in the brain, and are likely to have developed a health condition, such as diabetes, liver disease, or cancer. Normal physiological aging is associated with BBB disruption through multiple mechanisms including decreased expression of endothelial tight junction proteins, increased microglial activation, and neuronal senescence (Erdő et al., 2017). Collectively, this leads to high exposure levels in a brain that is less resilient or unable to compensate for functional changes resulting from chemical exposure (Erdő et al., 2017).

Beyond normal aging, any individuals with a compromised BBB may be more susceptible to neurotoxic damage by PFAS (Wang J. et al., 2018). Many neurological conditions, including Alzheimer’s disease (AD), Parkinson’s Disease (PD), multiple sclerosis (MS), Amyotrophic lateral sclerosis (ALS), Huntington’s disease, and brain tumors, confer heightened damage by PFAS and other toxicants by functionally compromising the BBB (Weiss et al., 2009; Erdő et al., 2017). In fact, the severity of symptoms for MS and AD have been shown to be tightly coupled with BBB integrity, with research indicating that onset and progression of symptoms correlates strongly with BBB disruption (Fabis et al., 2007; Morgan et al., 2007; Zlokovic, 2011). In this way, PFAS exposure could be more hazardous for individuals with pre-existing neurological conditions or a predisposition for these diseases, as PFAS can cause damage to the BBB, which could accelerate symptom onset or exacerbate symptom severity (Weiss et al., 2009; Wang J. et al., 2018). While research on the associations between PFAS exposure and neurodegenerative disorders is limited, one epidemiological study found a positive correlation between high PFAS concentrations in participant’s drinking water and relative risk for AD in deceased subjects of both sexes, and PD in females only (Mastrantonio et al., 2018). Another study found a correlation between increased PFOS and PFHxS exposure and downregulation of two microRNAs (miRNAS), miR-101-3p and miR-19a-3p, which have also been found to be downregulated in the blood and brain tissue of AD patients (Xu et al., 2020).

Diabetes is another condition that may confer increased risk to PFAS-induced toxicity in the brain, as it is also known to alter the structure and function of the BBB, primarily by hyper-glycemia induced disruption of brain microvasculature and oxidative stress (Prasad et al., 2014). In fact, Wang J. et al. (2018) reported that increased levels of glucose in serum samples from hospital patients were positively associated with increased penetration of PFOA and 6:2 chlorinated polyfluorinated ether sulfonate (6:2 Cl-PFESA) through the BBB. Three different studies have reported increased diabetes driven mortality in populations exposed to high concentrations of PFAS, including individuals with contaminated drinking water and fluorochemical factory workers (Leonard et al., 2008; Lundin et al., 2009; Mastrantonio et al., 2018). More studies are needed to fully appreciate the complex relationship between diabetic disease risk, onset, and/or exacerbation and PFAS exposure.

## Conclusion

Although there is considerable discrepancy across studies, the evidence suggests that PFAS can impact the nervous system, with particularly harmful effects from developmental exposures or exposures in sensitive populations. However, the limitations and inconsistencies in the current research make the severity of the neurotoxicological ramifications of PFAS exposure largely unknown. Most PFAS research is currently focused on PFOS and PFOA, while there are thousands of other compounds that receive very little research focus. While it would be impossible to thoroughly assess the neurotoxicological differences between PFAS compounds on an individual basis, grouping of PFAS compounds by chemical class is a necessary step towards understanding PFAS behavior in humans and wildlife more holistically (Cousins et al., 2020; Kwiatkowski et al., 2020). Additionally, while most experimental studies focus on short-term, very high dose PFAS exposures to single compounds, the most realistic exposures for humans and wildlife are mixtures exposures that are more chronic and low-dose in nature (Piekarski et al., 2020). In a review of the literature on PFAS exposure studies, Piekarski et al. found that the concentrations of PFAS measured in serum from the average animal study (∼94,996 ng/ml) was roughly 168 times the average level of PFAS in the serum of human populations with specific exposure concerns, approximately 564 ng/ml (Piekarski et al., 2020). Although the magnitude of these differences seems striking, there are legitimate biological justifications for the use of higher dose exposures in animal studies, including appreciable differences in PFAS elimination rates across species (Pizzurro et al., 2019). For instance, the elimination half-life for PFOS is estimated to be 3.3–5.4 years in humans, 110–200 days in monkeys, and only 24–83 days in rats (Pizzurro et al., 2019). It is also important to note that many PFAS congeners are not included in human exposure estimates. However, the paucity of experimental data representing chronic, low-dose PFAS exposures in animal studies is a discernable limitation in our understanding of PFAS health effects in the brain, as well as other organ systems. Finally, analysis of PFAS as neurotoxicants also needs to consider the state-dependency of the risk posed by these compounds and must place emphasis on investigation of populations that may be at a higher risk of experiencing the neurotoxicological impacts of PFAS exposure.
